# Construction and Validation of a Potent Epigenetic Modification-Related Prognostic Signature for Osteosarcoma Patients

**DOI:** 10.1155/2021/2719172

**Published:** 2021-11-22

**Authors:** Siyu Liu, Bing Wu, Xiaomin Li, Lulu Zhao, Wen Wu, Songtao Ai

**Affiliations:** Department of Radiology, Shanghai Ninth People's Hospital, Shanghai Jiao Tong University School of Medicine, Shanghai 200011, China

## Abstract

**Background:**

Increasing evidence has shown that tumorigenesis correlates with aberrant epigenetic factors, such as DNA methylation, histone modification, RNA m6A modification, RNA binding proteins, and transcription factors. However, it is unclear that how epigenetic genes linked with alteration contribute to osteosarcoma's incidence and clinical prognosis. We developed an epigenetic modification-related prognostic model that may improve the diagnosis and prognosis of osteosarcoma.

**Methods:**

We investigated the epigenetic modification-associated genes and their clinical significance in osteosarcoma in this research. Our gene transcriptome data were obtained from the TARGET database and the GEO database. Bioinformatics techniques were used to investigate their functionalities. The diagnostic and prognostic models were constructed using univariate and multivariate Cox regression. In addition, we developed a nomogram indicating the practicability of the prognostic model described above.

**Results:**

A risk score model constructed based on four epigenetic modification-related genes (MYC, TERT, EIF4E3, and RBM34) can effectively predict the prognosis of patients with osteosarcoma. Based on the risk score and clinical features, we constructed a nomogram.

**Conclusion:**

Epigenetic modification-related genes have been identified as important prognostic markers that may assist in osteosarcoma therapy therapeutic decision-making.

## 1. Introduction

The most frequent primary malignant bone tumor is osteosarcoma, which is primarily caused by primitive malignant bone mesenchymal cells [1, 2]. Osteosarcoma is most common in teenagers and young adults, with an annual incidence of around 4.4 per million [3, 4]. Patients with chemotherapy drug-resistant and lung metastatic osteosarcoma have a poor prognosis, with a 5-year survival rate of only 20% [5]. In addition, patients with the same clinical or pathological conditions receiving the same treatment protocol have different clinical outcomes due to their genetic heterogeneity [6]. As a result, in-depth investigation of the molecular processes underlying the development of osteosarcoma is critical to identifying useful prognostic biomarkers to assist patient risk stratification, which fits with the precision medicine approach.

High-throughput sequencing technologies, gene chips, and large-scale RNA-seq transcriptome sequencing have been widely used to identify genes associated with various cancers, elucidate carcinogenesis, and improve cancer treatment [7, 8]. Osteosarcoma has received a great deal of attention recently, including the use of biomolecules and risk models to assess the prognosis [9, 10]. These methods, however, have not yet been used in clinical practice because of insurmountable obstacles including the possibility of overfitting owing to limited sample sizes [11]. Epigenetics is a concept that refers to dynamic and heritable alterations in different DNA sequences [12]. Abnormalities in epigenetics may affect expression regulation and influence the balance of expression of oncogenes, leading to tumor development [13]. The major epigenetic alterations in cancer development include methylation of RNA, including m6A modifications of RNA and histone modifications, which are considered to be the most important factors in cancer development [14]. Previous studies have revealed the functions of specific epigenetically related genes but have been deficient in their examination of the whole of this complex system. Also, the diagnostic and prognostic significance of these genes in the treatment of osteosarcoma remains uncertain. We identified five different types of epigenetic modification-related genes (EMGs) in the current study, totaling 2397 genes, including m6A modifications of RNA, histone modifications, DNA methylation modifications, RNA binding proteins, and transcription factors [15–19]. We obtained mRNA expression profiles and clinical data from TCGA and GEO databases for patients with osteosarcoma. We used differential gene expression linked to epigenetic alteration to build a predictive signature in the TARGET cohort, and we verified the model's stability and reliability in the GEO cohort. Then, to investigate its possible mechanism, we performed a functional enrichment study.

## 2. Materials and Methods

### 2.1. Data Processing

We obtained RNA-seq data and clinical features from TARGET database for 88 patients with osteosarcoma. Our inclusion criteria for patients were as follows: (1) histologically diagnosed with osteosarcoma; (2) available expression profilings; and (3) overall survival time greater than 30 days. We also downloaded RNA-seq data from the GTEx database for 396 normal skeletal muscle samples. We selected the dataset from the GEO database (ID: GSE21257) to be used as the validation dataset. Epigenetic modification-related genes were referred to as EMGs according to previous studies, including m6A-related genes, histone modification-related genes, RNA-binding proteins, transcription factors, and DNA methylation enzymes (Table S1). The R package “limma” was used to perform differential expression analysis of transcriptome data. The thresholds for differential genes (DEGs) were set at *P* value <0.05 and |log_2_FC| >0.5.

### 2.2. GO and KEGG Analysis

For the biological activities of these differentially expressed EMGs, GO and KEGG analyses were used to thoroughly study the functions. The “clusterProfiler” program in R software was used to classify genes. Functional enrichment studies for GO keywords and KEGG pathways were carried out using a hypergeometric distribution with a significance level of *P* < 0.05.

### 2.3. Developing a PPI Network and Module Screening

We uploaded EMGs with differential expression to the STRING database to analyze protein-protein interactions. The PPI network was further constructed and displayed using the Cytoscape 3.8.0 program. The MCODE was used to analyze the relevant modules and genes of the PPI network, and the number of nodes was required to be greater than 5. *P* < 0.05 was considered a significant difference.

### 2.4. Prognostic Model Construction

By using the R package survival, we were able to perform a univariate Cox regression analysis on the different EMGs in the training dataset. To explore the potential important genes, a log-rank test was conducted. Subsequently, we constructed a prognostic model by multivariate Cox regression analysis and generated a risk score to assess the patients' prognosis using the significant candidate genes screened. The following formula was used to calculate the risk score for each sample:

(1) $$\begin{array}{c} \text{risk score}=\beta_{1}*{\text{Exp}}_{1}+\beta_{2}*{\text{Exp}}_{2}+\beta_{n}*{\text{Exp}}_{n}, \end{array}$$

where *β* represents the coefficient and Exp represents the amount of gene expression. Based on the median value of the risk score, we divided the osteosarcoma patients into a low-risk group and a high-risk group. The difference in overall survival (OS) between the two groups was observed using the log-rank test. We then performed ROC analysis using the software package surviveROC to assess the predictive power of our prognostic model. Finally, we selected a sample of 53 osteosarcoma patients in GSE21257, a dataset containing prognostic information, as a validation to verify the predictive power of this prognostic model.

### 2.5. Establishment of a Predictive Nomogram

We constructed a nomogram based on risk score and other clinical characteristics to provide clinicians with a tool to predict 1-, 3-, and 5-year survival rates for patients with osteosarcoma, and we also assessed the agreement between predicted values and observed patient information by calibration curves.

### 2.6. Gene Set Enrichment Analysis

We used Gene Set Enrichment Analysis (GSEA) [20] in the TARGET dataset to examine differences between high- and low-risk patients identified by prognostic models consisting of EMGs. Gene sets with an FDR less than 0.25 and a normalized *P* value less than 0.05 were deemed significant.

### 2.7. Chemotherapy Sensitivity Analysis

We evaluated NCI-60 by using the CellMiner database (https://discover.nci.nih.gov/cellminer) [21], which includes 60 different cancer cell lines from nine different malignancies. We investigated the relationship between the expression of EMGs in the model and drug sensitivity by using Pearson's correlation analysis.  Table S2 shows 263 drugs licensed by the FDA or in clinical development.

### 2.8. Statistical Analysis

For statistical methods, we utilized R software version 4.0.2 and multiple R packages, with a two-tailed *P* value of 0.05 indicating statistical significance. We performed univariate and multifactorial Cox regression analyses using the survival package. The survival package was used to create Kaplan–Meier analyses and to plot survival curves. Nomogram and calibration curves were done using the “rms” program. ROC curves were plotted over time using the “timeROC” software.

## 3. Results

### 3.1. EMGs with Differential Expression Found in Osteosarcoma Patients

**Figure 1:**
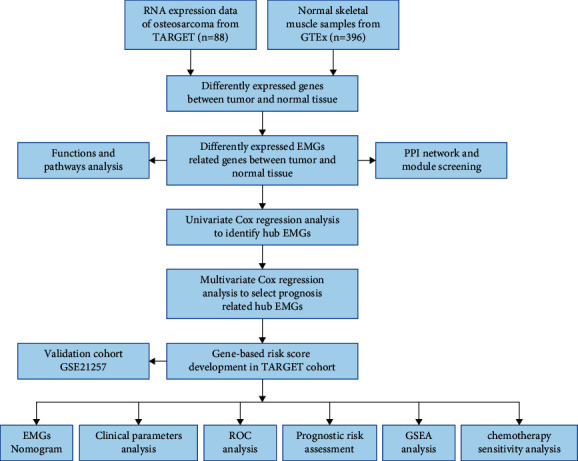
Whole procedures for analyzing EMGs in osteosarcoma.

**Figure 2:**
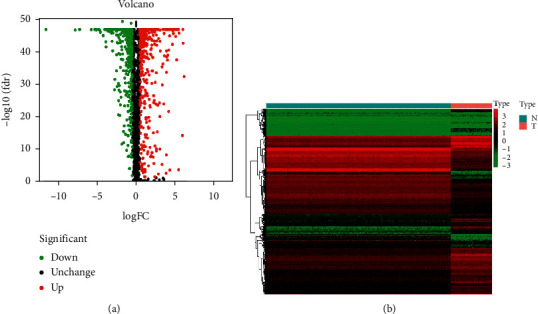
The differentially expressed EMGs in osteosarcoma. (a) Heat map. (b) Volcano plot.

We used various sophisticated computational techniques to perform a comprehensive examination of the essential functions and prognostic values of EMGs in osteosarcoma.  Figure 1 depicts the research design. The osteosarcoma datasets obtained from TARGET included 88 tumor samples, while the GTEx databases had 396 normal tissue samples. A total of 2397 EMGs were included in the analysis, with 867 EMGs meeting the study's screening criteria (*P* < 0.05 and |log2FC| > 0.5), consisting of 454 upregulated and 413 downregulated EMGs.  Figure 2 shows the expression of these different EMGs.

### 3.2. GO and KEGG Analysis of EMGs with Different Expression Levels

**Figure 3:**
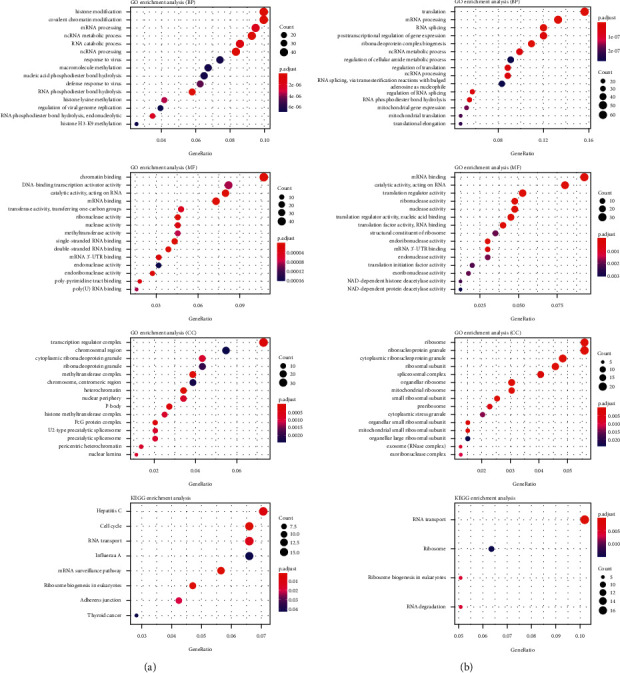
KEGG pathway and GO enrichment analysis of aberrantly expressed EMGs. (a) Upregulated EMGs. (b) Downregulated EMGs.

To investigate the functions and possible mechanisms of these EMGs, we classified them as up- or downregulated according to their expression. We next utilize these differentially expressed EMGs to perform functional enrichment analysis. According to the findings, downregulated differentially expressed EMGs were substantially enriched in the mRNA, ncRNA modification, and processing associated pathways. Differentially expressed EMGs that were upregulated were substantially enriched in RNA splicing and mRNA processing-associated pathways. RNA transport was shown to be abundant in both upregulated and downregulated differentially expressed EMGs during KEGG analysis.  Figure 3 shows further information.

### 3.3. PPI Network Construction and Identification of Key Modules

**Figure 4:**
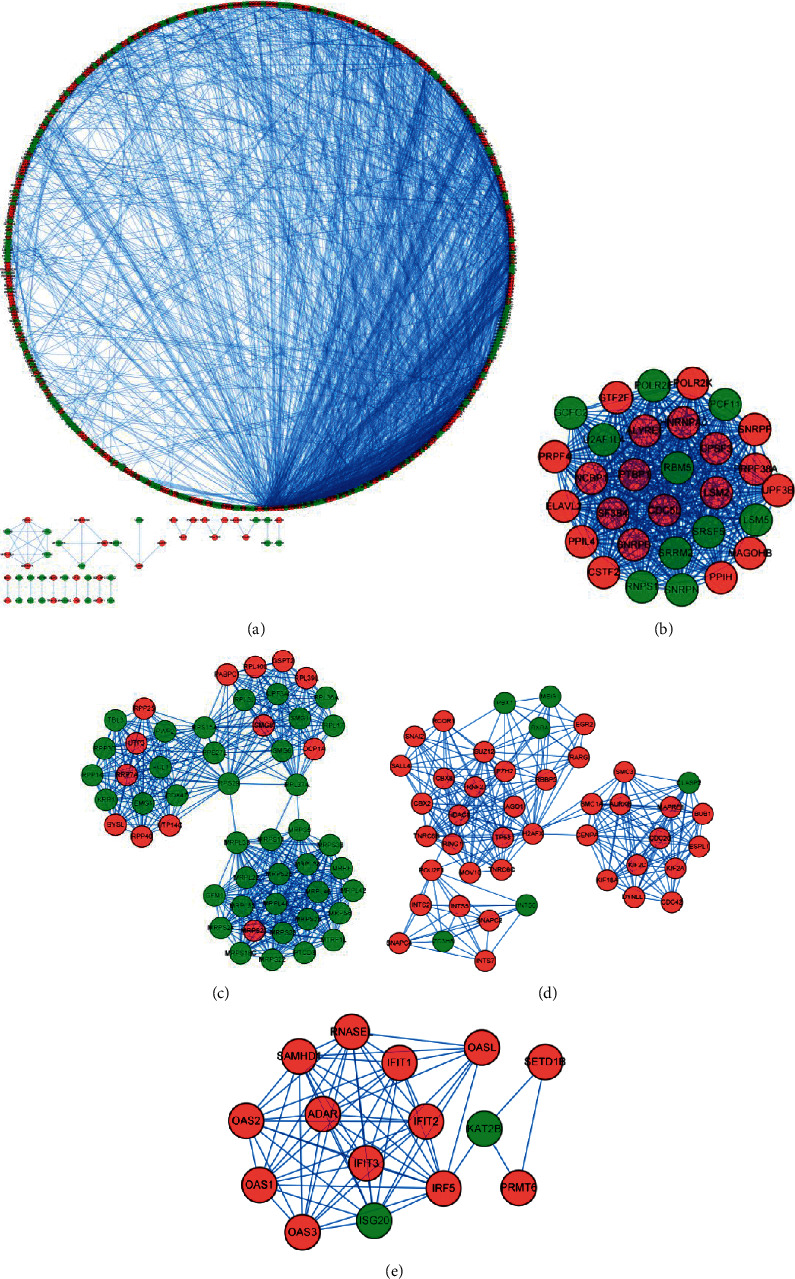
PPI network construction and identification of key modules. (a) PPI network of differently expressed EMGs; (b–d) critical module from the PPI network.

We used Cytoscape software to analyze a PPI network with 531 nodes and 2941 edges constructed from the STRING database [22] (Figure 4(a)) to investigate the function of differentially expressed EMGs in osteosarcoma. We identified the four most important modules by Cytoscape software. Module 1 had 30 nodes and 423 edges (Figure 4(b)). Module 2 had 52 nodes and 486 edges (Figure 4(c)). Module 3 had 44 nodes and 269 edges (Figure 4(d)). Module 4 had 15 nodes and 67 edges (Figure 4(e)).

### 3.4. Prognosis-Related Hub Epigenetic Modification-Related Genes

After identifying 867 differentially expressed EMGs genes, we calculated the association between differentially expressed EMGs and OS by univariate Cox regression analysis and Kaplan–Meier method, and the results of the study showed that 53 candidate EMGs genes were significantly associated with OS (Table S3). Following this, the effect of these 53 potential hub EMGs on OS was investigated using multivariate Cox analysis, which revealed that four hub EMGs were independent prognostic indicators for osteosarcoma patients (Table 1).

### 3.5. Construction and Validation of the Prognostic Model

**Figure 5:**
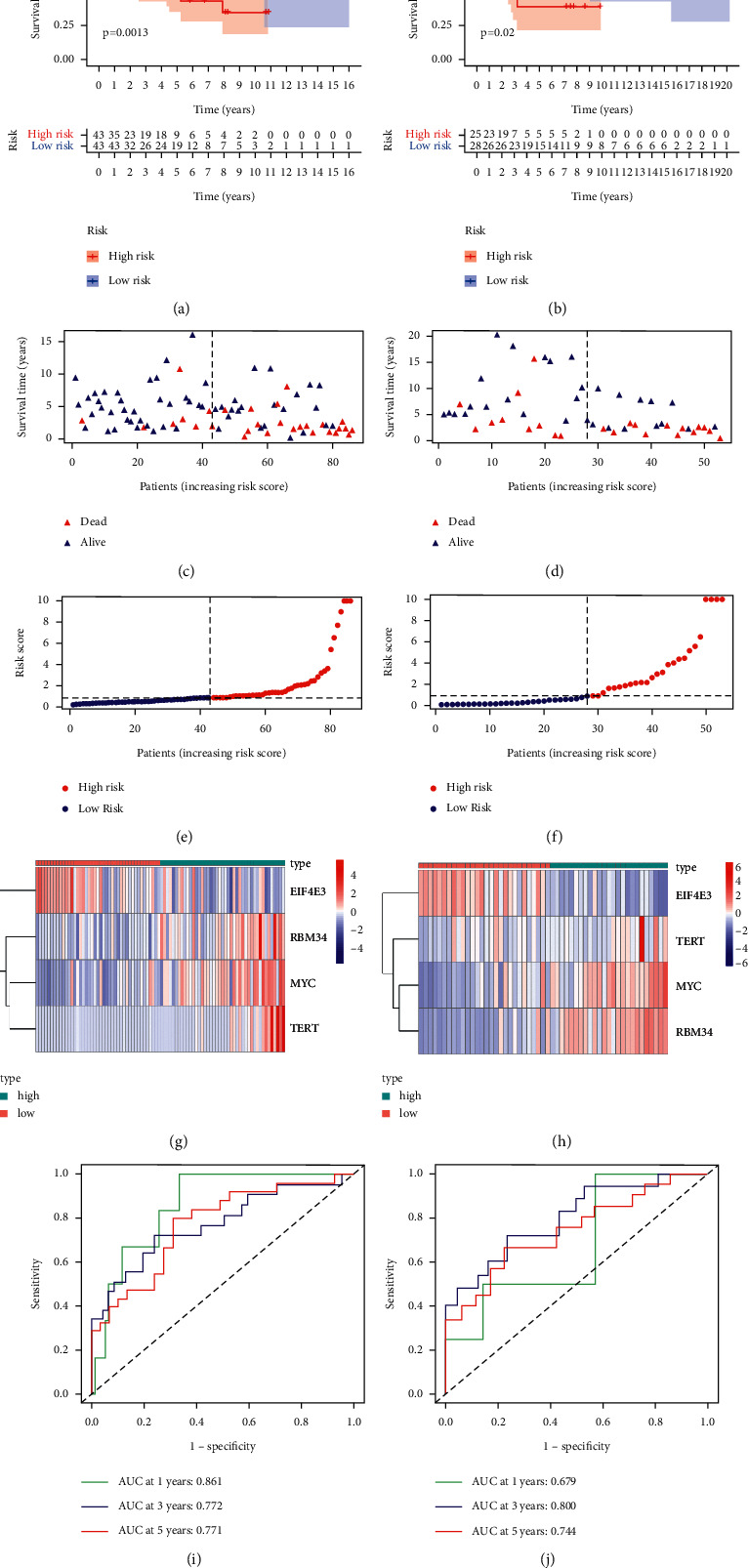
Risk score analysis of four-genes prognostic model in TARGET and GEO cohort. (a), (c), (e) (g), and (i) represent TARGET cohort; (b), (d), (f), and (h) represent GEO cohort. (a, b) Survival curve for low- and high-risk groups. (c, d) Survival status of low- and high-risk groups. (e, f) The mean value and distribution of the risk score. (g, h) Expression heat map. (i, j) ROC curves.

We then constructed a prognostic model based on the four key EMGs using the previously described method. And, a survival analysis was performed to analyze its predictive power. Based on the median risk score, the 88 osteosarcoma patients were divided into two groups: a low-risk group and a high-risk group. The results showed that the high-risk group had a lower overall survival rate than the low-risk group (Figure 5(a)). We further analyzed the predictive power of the markers comprising these four EMGs by means of a time-dependent ROC analysis. The area under the ROC curve (AUC) for this EMGs risk score was 0.861 after one year, 0.772 after three years, and 0.771 after five years (Figure 5(i)), indicating that it has a good diagnostic performance. Figures 5(c), 5(e), and 5(g) show the gene expression heatmap, patient survival, and risk score of the prognostic model consisting of four EMGs in the low-risk and high-risk groups. Subsequently, we performed the same analysis on the GSE21257 dataset to analyze whether the prediction model consisting of the four EMGs had the same predictive performance in the cohort of osteosarcoma patients. In the GSE21257 dataset, the results showed that patients in the high-risk group had a worse OS than patients in the low-risk group (Figures 5(b), 5(d), 5(f), 5(h), and 5(j)). These findings above suggest that our prognostic model has high sensitivity and specificity.

### 3.6. The Prognostic Model Risk Score and Clinical Features

**Figure 6:**
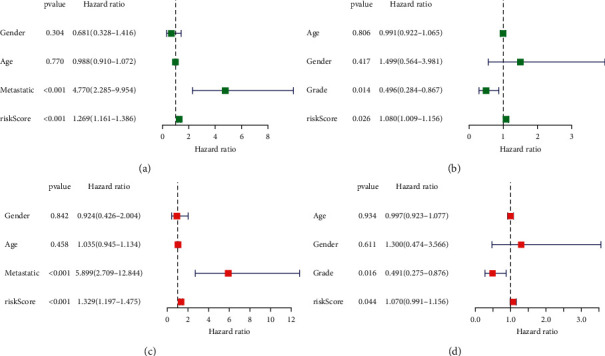
Cox regression analyses for the risk score. Univariate and multivariate analysis for (a, c) TARGET dataset; (b, d) GEO dataset.

To understand whether our risk score might predict the prognosis of patients with osteosarcoma independently of other clinical characteristics, we subsequently performed a multivariate Cox regression analysis including age, sex, metastasis, and risk score in the TARGET dataset and age, sex, grade, and risk score in the GEO dataset.  Figure 6 shows that our risk scores can predict patient prognosis independent of other clinical characteristics.

**Figure 7:**
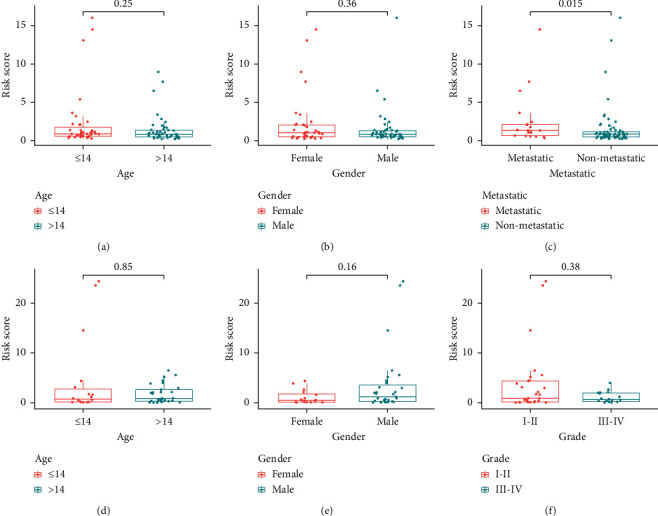
The risk score in two groups divided by clinical characteristics. (a–c) TARGET dataset; (d–f) GEO dataset.

We analyzed the relationship between risk scores and clinical characteristics of patients with osteosarcoma in the TARGET dataset and found that risk scores were substantially higher in patients in the metastatic group than in those in the nonmetastatic group (Figure 7(c)). However, there were no significant differences in age and gender in the TARGET dataset (Figures 7(a) and 7(b)). However, there were differences in the GEO dataset (Figures 7(d)–7(f)).

### 3.7. Building a Predictive Nomogram

**Figure 8:**
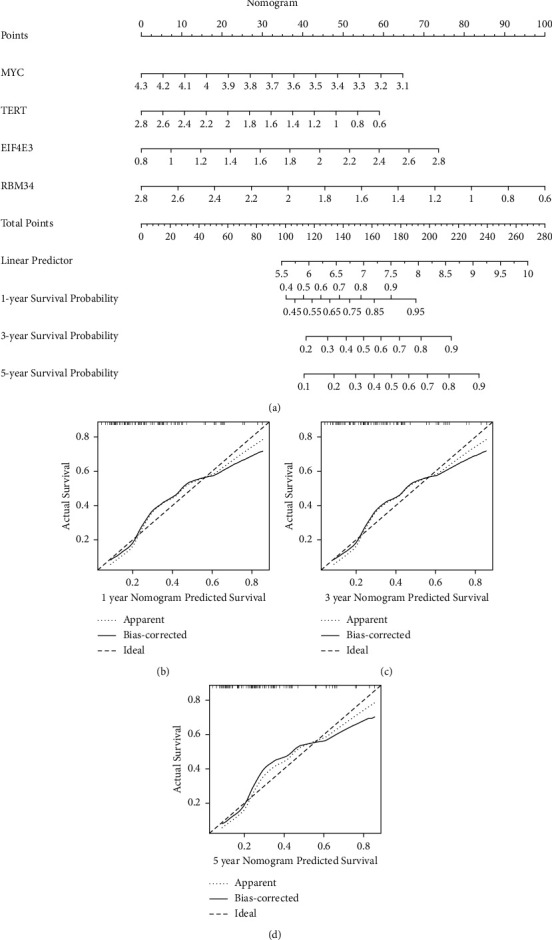
Nomogram for predicting OS of osteosarcoma patients in TARGET cohort. (a) Predictive nomogram. (b–d) Calibration curve.

The four discovered hub EMGs were then used to create a nomogram, which allowed physicians to assess the survival of patients with osteosarcoma (Figure 8). By using the score table in the nomogram, each of the included variables was scored based on the previous results. The scores of all included factors were then summed to obtain the total patient score, and the predicted 1-, 3-, and 5-year survival rates for each patient were obtained based on the total score.

### 3.8. GSEA Analysis

**Figure 9:**
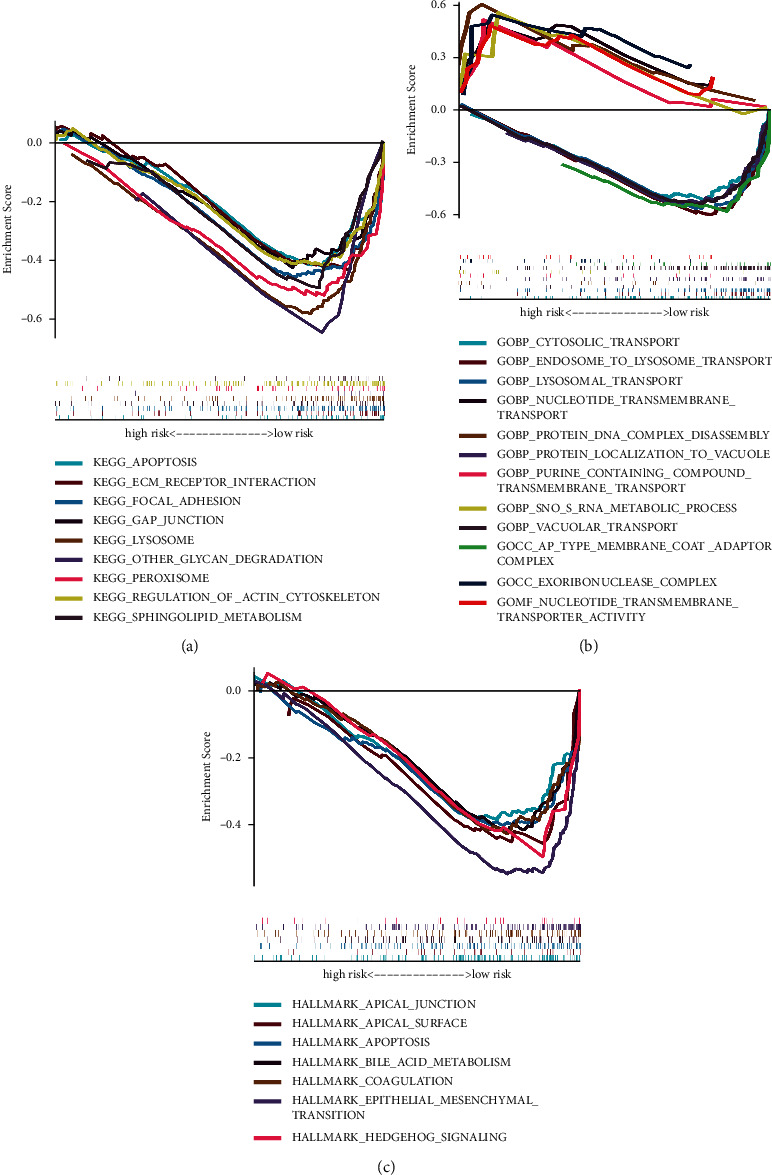
GSEA analysis. (a) KEGG, (b) GO, and (c) Hallmark gene set.

We used the GSEA method to compare the enrichment of GO and KEGG between the high-risk and low-risk groups. Based on the notation derived from GO functional enrichment analysis, cytosolic transport and endosome to lysosome transport were substantially enriched in the low-risk group (Figure 9(b)). We also found nine KEGG pathways were enriched in the high-risk group at an FDR of 0.05 (Figure 9(a)). By using the Hallmarks gene set from the TCGA dataset to perform GSEA analysis, it was obtained that apical junction and apoptosis were statistically significant, similar to the results obtained for KEGG (Figure 9(c)).

### 3.9. Expression of Genes with Prognostic Efficacy and Sensitivity of Cancer Cells to Chemotherapy

**Figure 10:**
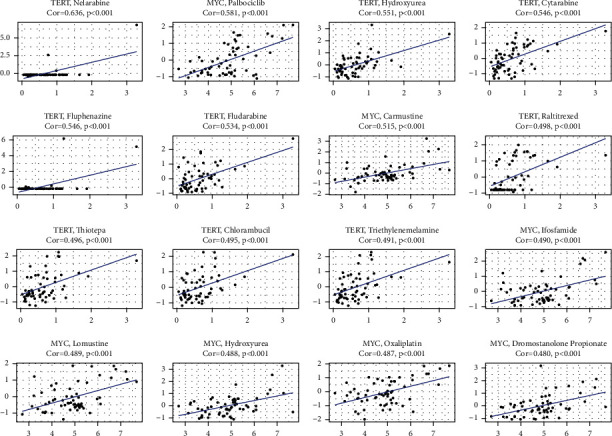
Relationship between the prognostic EMGs and drug sensitivity.

To discover genes predictive of drug sensitivity, we examined the expression of these genes in NCI-60 cell lines and observed how they correlate with drug sensitivity. TERT and MYC were strongly correlated with chemotherapeutic drug sensitivity, as shown by the findings, which were statistically significant (*P* < 0.01) (Figure 10). TERT and MYC, for example, have been linked to enhanced drug sensitivity of cancer cells to nelarabine, palbociclib, hydroxyurea, cytarabine, fluphenazine, fludarabine, carmustine, and other drugs.

## 4. Discussion

Although many advances have been made in diagnosis and treatment in recent decades, patients with osteosarcoma still have low survival rate. Future research should be aimed at discovering genes that have prognostic value. Currently, there are few biomarkers with high sensitivity for patients with osteosarcoma. And, in past studies, bioinformatic studies were often limited to a single database or single gene prognostic value, and this approach has limitations. Many elements of epigenetic alteration controlling gene expression that interfere with tumor development have been discovered by scientists in recent decades [23, 24]. Today's hot topics in oncology research include DNA methylation, m6A modification of RNA, and histone modification. Previous studies focused on the prognosis and function of individual epigenetically related genes. By using bioinformatics methods of analysis, we screened genes that can be used in the prediction of osteosarcoma patients' prognosis and performed related genes function prediction analysis, which will help future experimental validation and research. In addition, epigenetic regulation-related genes regulate gene expression through RNA-binding proteins and transcription factors; therefore, we collected five types of EMGs. By analyzing these EMGs, we constructed a prognostic model that can accurately predict osteosarcoma patients' prognosis and validated the model using another dataset.

In our study, 2397 epigenetic modification-related genes in normal samples from the GTEx dataset and osteosarcoma samples from the TARGET dataset were analyzed, and we obtained 867 DEGs. On the other hand, using Cox regression analysis, we found that 53 DEGs were associated with OS. We further constructed a prognostic model consisting of 4 genes validated in the GEO dataset. Our study found that high-risk group patients had more metastases and shorter OS time than in the low-risk group. Also, we obtained after statistical analysis that our risk score is predictive of patient survival independently of other clinical information. We also found high expression of certain EMGs were associated with increased resistance to some FDA-approved chemotherapeutic agents, and these results suggest that targeting tumor resistance genes may hold therapeutic promise for patients in the high-risk group.

This study's prognostic model was made up of four epigenetic modification-related genes (MYC, TERT, EIF4E3, and RBM34). MYC is one of the most extensively studied cancer-causing genes, having been linked to the development, maintenance, and advancement of a variety of cancers [25–27]. Gene amplification, chromosomal translocations, activation of superenhancers, changes in cell signaling, altered protein degradation, and mutations are among the mechanisms that cause these changes [28–30]. TERT is typically active only during early embryonic development and in cells with high proliferative capacity, whereas it is dormant in the majority of somatic cells in adults. TERT, on the other hand, is reactivated in most malignancies, and by lengthening telomeres, it contributes to cancer development and progression. TERT is one of the two main components of the larger telomerase complex, which adds particular short repetitive DNA sequences to telomeres to lengthen them. EIF4E is a powerful oncogene that is found in about 30% of human malignancies [31, 32]. EIF4E participates in mRNA export and translation by binding the methyl 7 guanosine cap present on the 5′ end of mRNAs. Typically, these transcripts encode proteins associated with proliferation, survival, invasion, and metastasis [33, 34]. The RNA-binding protein RBM34 has been shown to be overexpressed in recurrent prostate cancer [35].

To exploit the underlying mechanism of the signature, we carried out the GSEA method. The results indicated that the high-risk patients were mainly involved in tumor development by activating cell apoptosis, gap junction, and epithelial-mesenchymal transition (EMT). In terms of biological process, the gene sets were mainly enriched in lysosomal transport and endosome to lysosome transport, suggesting that patients in the high-risk group might regulate tumor progression in the autophagy pathway.

To the best of our knowledge, this is the first research to develop a predictive signature in osteosarcoma based on epigenetically modified genes. Our study, however, had some limitations. Further confirmation of the signature's efficacy in additional independent prospective trials and functional tests of the identified genes is required in this research. Beyond this, we need more prospective clinical studies and larger sample sizes to assess the diagnostic performance of the prognostic model. As a result, there is still more work to be done before the results can be applied to clinical practice.

## 5. Conclusion

A new epigenetically modified-related gene signature was created, and it demonstrated significant clinical utility in predicting the OS of patients with osteosarcoma. The signature may serve as a reliable biomarker for the early detection and prognosis of osteosarcoma.

## Figures and Tables

**Table 1:** Four epigenetic modification-related genes significantly associated with OS in the TARGET cohort.

Gene symbol	Coef	HR	HR.95L	HR.95H	*P* value
MYC	1.693788	5.440048	1.192569	24.81544	0.028714
TERT	1.101408	3.008399	1.320102	6.855882	0.008773
EIF4E3	−0.90781	0.403405	0.115026	1.414777	0.026192
RBM34	1.057254	2.878456	1.068718	7.752752	0.036489

## Data Availability

The datasets analyzed in this study are available in Therapeutically Applicable Research to Generate Effective Treatments (TARGET), Gene Expression Omnibus (GEO), and Genotype Tissue Expression (GTEx).

## Abbreviations

DEGs: Differentially expressed genes
TARGET: Therapeutically applicable research to generate effective treatments
GTEx: Genotype Tissue Expression
GEO: Gene Expression Omnibus
GO: Gene Ontology
KEGG: Kyoto Encyclopedia of Genes and Genomes
GSEA: Gene set enrichment analyses
FDR: False discovery rate.

## Supplementary Materials

Supplementary Materials
*Supplementary File Table S1*. Epigenetic modification-related genes based on previous literature and databases. *Supplementary File Table S2*. 263 drugs approved by FDA or in clinical trials. *Supplementary File Table S3*. 53 candidate hub EMGs associated with OS through the univariate Cox regression analysis.
