# Supplementary material for: Construction and Validation of a Potent Epigenetic Modification-Related Prognostic Signature for Osteosarcoma Patients
Source: J Oncol. 2021 Nov 22;2021:2719172. doi: 10.1155/2021/2719172 (PMC8629625; doi:10.1155/2021/2719172)
Supplement: Supplementary Materials — Supplementary File Table S1. Epigenetic modification-related genes based on previous literature and databases. Supplementary File Table S2. 263 drugs approved by FDA or in clinical trials. Supplementary File Table S3. 53 candidate hub EMGs associated with OS through the univariate Cox regression analysis. [file 2719172.f1.zip › 2719172.f1/Table S1.docx]

Table S1. Epigenetic modification related genes based on previous literature and databases.

| Transcription factor | M6A-related gene | Histone modification-related genes | RNA binding protein | DNA methylase |
| --- | --- | --- | --- | --- |
| ADNP | METTL14 | ASH1L | A1CF | DNMT3A |
| AFF4 | METTL16 | ATM | AAR2 | DNMT1 |
| AR | RBM15 | ATR | AARS | DNMT3B |
| ARID3A | VIRMA | AURKB | AARS2 | DNMT3L |
| ARNT | WTAP | BAZ1B | AARSD1 | TET1 |
| ARNTL | ZC3H13 | BRCC3 | ABCE1 | TET2 |
| ASCL1 | FTO | BUB1 | ABCF1 | TET3 |
| ASH2L | HNRNPA2B1 | C14orf169 | ABCF2 |  |
| ATF1 | HNRNPC | CARM1 | ABCF3 |  |
| ATF2 | IGF2BP1 | CDK17 | ABL2 |  |
| ATF3 | YTHDC1 | CDK3 | ABT1 |  |
| ATF4 | YTHDC2 | CDK5 | AC004381.6 |  |
| ATF7 | YTHDF1 | CDY1B | ACIN1 |  |
| BACH1 | YTHDF2 | CDYL | ACLY |  |
| BACH2 | YTHDF3 | CHUK | ACO1 |  |
| BATF | IGF2BP2 | CLOCK | ACTBL2 |  |
| BCL11A | IGF2BP3 | CREBBP | ACTN1 |  |
| BCL3 | ALKBH5 | DAPK3 | ADAD1 |  |
| BCL6 | MELLT3 | DOT1L | ADAD2 |  |
| BDP1 |  | DTX3L | ADAR |  |
| BHLHE40 |  | DUSP1 | ADARB1 |  |
| BMI1 |  | EHMT1 | ADARB2 |  |
| BRCA1 |  | EHMT2 | ADAT1 |  |
| BRD1 |  | ELP3 | ADAT2 |  |
| BRD2 |  | EP300 | ADAT3 |  |
| BRD3 |  | EYA1 | AEN |  |
| BRD4 |  | EYA2 | AFF1 |  |
| BRF1 |  | EYA3 | AFF2 |  |
| BRF2 |  | EZH1 | AFF3 |  |
| C17orf96 |  | EZH2 | AFF4 |  |
| CBFB |  | GSG2 | AGFG1 |  |
| CBX2 |  | GSK3B | AGO1 |  |
| CBX3 |  | GTF3C4 | AGO2 |  |
| CBX5 |  | HAT1 | AGO3 |  |
| CBX7 |  | HDAC1 | AGO4 |  |
| CBX8 |  | HDAC10 | AHNAK2 |  |
| CDK2 |  | HDAC11 | AIMP1 |  |
| CDK7 |  | HDAC2 | AIMP2 |  |
| CDK8 |  | HDAC3 | AKAP1 |  |
| CDK9 |  | HDAC4 | AKAP13 |  |
| CDX2 |  | HDAC5 | AKAP17A |  |
| CEBPA |  | HDAC6 | AKAP2 |  |
| CEBPB |  | HDAC7 | AKAP8 |  |
| CENPA |  | HDAC8 | AKAP8L |  |
| CHD1 |  | HDAC9 | AKNA |  |
| CHD2 |  | HLCS | AKR1B1 |  |
| CHD7 |  | HUWE1 | AL844220.1 |  |
| CHD8 |  | JAK2 | ALAS2 |  |
| CIITA |  | JHDM1D | ALKBH1 |  |
| CPSF3L |  | JMJD5 | ALKBH5 |  |
| CREBBP |  | JMJD6 | ALKBH8 |  |
| CTNNB1 |  | KAT2A | ALX4 |  |
| CUX1 |  | KAT2B | ALYREF |  |
| DNMT1 |  | KAT5 | ANG |  |
| DNMT3A |  | KDM1A | ANGEL1 |  |
| DYRK1A |  | KDM1B | ANGEL2 |  |
| E2F1 |  | KDM2A | ANK3 |  |
| E2F3 |  | KDM2B | ANKHD1 |  |
| E2F4 |  | KDM3A | ANKRD17 |  |
| E2F6 |  | KDM3B | ANKRD27 |  |
| E2F7 |  | KDM4A | ANPEP |  |
| EBF1 |  | KDM4B | ANXA1 |  |
| EED |  | KDM4C | ANXA11 |  |
| EGR1 |  | KDM4D | ANXA7 |  |
| EGR2 |  | KDM5A | AP1G1 |  |
| EHF |  | KDM5B | APBA3 |  |
| EHMT2 |  | KDM5C | APEX1 |  |
| ELF1 |  | KDM5D | API5 |  |
| ELF5 |  | KDM6A | APOBEC1 |  |
| ELK1 |  | KDM6B | APOBEC2 |  |
| ELK4 |  | LIMK2 | APOBEC3B |  |
| ELL2 |  | MAP3K8 | APOBEC3F |  |
| EMX1 |  | MASTL | APOBEC3G |  |
| EOMES |  | MGEA5 | APOBEC3H |  |
| EP400 |  | MLL | APOBEC4 |  |
| EPAS1 |  | MLL2 | APTX |  |
| EPO |  | MLL3 | AQR |  |
| ERCC6 |  | MLL5 | ARHGAP36 |  |
| ERG |  | MYSM1 | ARHGAP5 |  |
| ESR1 |  | MYST1 | ARHGAP9 |  |
| ESRRA |  | MYST2 | ARHGEF28 |  |
| ETS1 |  | MYST3 | ARL6IP4 |  |
| ETV1 |  | MYST4 | ASCC1 |  |
| EZH1 |  | NAT10 | ASCC3 |  |
| EZH2 |  | NCOA1 | ASH1L |  |
| FLI1 |  | NCOA3 | ASNA1 |  |
| FOS |  | NEK6 | ASPH |  |
| FOSL1 |  | NEK9 | ATF7 |  |
| FOSL2 |  | NSD1 | ATL3 |  |
| FOXA1 |  | PADI4 | ATP13A1 |  |
| FOXA2 |  | PAK2 | ATP6AP1 |  |
| FOXK1 |  | PARG | ATXN1 |  |
| FOXM1 |  | PARP1 | ATXN10 |  |
| FOXO1 |  | PHF8 | ATXN1L |  |
| FOXO3 |  | PKN1 | ATXN2 |  |
| FOXP1 |  | PPM1D | ATXN2L |  |
| FOXP2 |  | PPP1CC | AUH |  |
| FOXP3 |  | PPP2CA | AVL9 |  |
| GABPA |  | PPP2CB | AZGP1 |  |
| GATA2 |  | PPP4C | BAG3 |  |
| GATA3 |  | PPP5C | BARD1 |  |
| GATA4 |  | PRDM2 | BASP1 |  |
| GATA6 |  | PRDM9 | BAZ2A |  |
| GATAD1 |  | PRKCB | BAZ2B |  |
| GREB1 |  | PRKCD | BCDIN3D |  |
| GRHL2 |  | PRKDC | BCLAF1 |  |
| GTF2B |  | PRMT1 | BICC1 |  |
| GTF2F1 |  | PRMT2 | BLVRB |  |
| GTF2I |  | PRMT5 | BMS1 |  |
| H2AFX |  | PRMT6 | BOLL |  |
| HCFC1 |  | PRMT7 | BOP1 |  |
| HDAC1 |  | PRMT8 | BRCA1 |  |
| HDAC2 |  | RAG1 | BRIX1 |  |
| HDAC3 |  | RBX1 | BSG |  |
| HDAC6 |  | RING1 | BSPRY |  |
| HEY1 |  | RNF168 | BUD13 |  |
| HIF1A |  | RNF2 | BYSL |  |
| HIRA |  | RNF20 | BZW1 |  |
| HNF1B |  | RNF40 | BZW2 |  |
| HNF4A |  | RNF8 | C11orf57 |  |
| HNF4G |  | RPS6KA4 | C11orf58 |  |
| HOXA9 |  | RPS6KA5 | C11orf67 |  |
| HOXB13 |  | SETD1A | C11orf68 |  |
| HOXB7 |  | SETD1B | C11orf80 |  |
| HOXC11 |  | SETD2 | C12orf43 |  |
| HOXC9 |  | SETD7 | C12orf65 |  |
| HSF1 |  | SETD8 | C13orf26 |  |
| HSF2 |  | SETDB1 | C16orf42 |  |
| IKZF1 |  | SETDB2 | C17orf37 |  |
| IRF1 |  | SETMAR | C17orf85 |  |
| IRF3 |  | SIRT1 | C19orf29 |  |
| IRF4 |  | SIRT2 | C19orf43 |  |
| IRF5 |  | SIRT3 | C19orf53 |  |
| JARID2 |  | SIRT6 | C1D |  |
| JMJD1C |  | SMYD2 | C1orf144 |  |
| JMJD6 |  | SMYD3 | C1orf55 |  |
| JUN |  | STK10 | C1QBP |  |
| JUNB |  | STK4 | C20orf27 |  |
| JUND |  | SUV39H1 | C2orf15 |  |
| KAT2B |  | SUV39H2 | C4orf29 |  |
| KAT5 |  | SUV420H1 | C4orf43 |  |
| KDM1A |  | SUV420H2 | C6orf170 |  |
| KDM2B |  | TLK1 | C7orf30 |  |
| KDM3A |  | UBR2 | C8orf33 |  |
| KDM4C |  | UHRF1 | C8orf59 |  |
| KDM5A |  | USP16 | C9orf114 |  |
| KDM5B |  | USP22 | C9orf129 |  |
| KDM5C |  | USP3 | C9orf23 |  |
| KDM6B |  | WHSC1 | C9orf6 |  |
| KLF11 |  | WHSC1L1 | C9orf78 |  |
| KLF4 |  | CDY1 | C9orf80 |  |
| KLF5 |  |  | CACTIN |  |
| LEF1 |  |  | CACYBP |  |
| LHX2 |  |  | CAD |  |
| LIN9 |  |  | CADM1 |  |
| LMNA |  |  | CALR |  |
| LMNB1 |  |  | CALR3 |  |
| LMO2 |  |  | CAND1 |  |
| LYL1 |  |  | CANX |  |
| MAF |  |  | CAPG |  |
| MAFF |  |  | CAPRIN1 |  |
| MAFK |  |  | CAPRIN2 |  |
| MAX |  |  | CARHSP1 |  |
| MAZ |  |  | CARS |  |
| MBD2 |  |  | CARS2 |  |
| MBD3 |  |  | CASC3 |  |
| MECP2 |  |  | CASK |  |
| MED12 |  |  | CBFA2T2 |  |
| MEF2A |  |  | CBX8 |  |
| MEF2B |  |  | CC2D1A |  |
| MEF2C |  |  | CCAR1 |  |
| MEIS1 |  |  | CCAR2 |  |
| MITF |  |  | CCDC114 |  |
| MXI1 |  |  | CCDC129 |  |
| MYB |  |  | CCDC43 |  |
| MYBL2 |  |  | CCDC59 |  |
| MYC |  |  | CCDC86 |  |
| MYH11 |  |  | CCDC87 |  |
| NANOG |  |  | CCDC88C |  |
| NCAPG |  |  | CCNC |  |
| NCOR2 |  |  | CCNT1 |  |
| NFATC1 |  |  | CCNT2 |  |
| NFE2 |  |  | CCRN4L |  |
| NFIC |  |  | CCT3 |  |
| NFYA |  |  | CCT5 |  |
| NFYB |  |  | CCT8 |  |
| NIPBL |  |  | CD200 |  |
| NOTCH1 |  |  | CD2BP2 |  |
| NR1H2 |  |  | CD3EAP |  |
| NR2C2 |  |  | CD55 |  |
| NR2F1 |  |  | CDC20 |  |
| NR2F2 |  |  | CDC37 |  |
| NR3C1 |  |  | CDC40 |  |
| NR4A1 |  |  | CDC42 |  |
| NR5A2 |  |  | CDC5L |  |
| NRF1 |  |  | CDHR3 |  |
| OGT |  |  | CDK11A |  |
| PAF1 |  |  | CDK5RAP1 |  |
| PAX3 |  |  | CDK9 |  |
| PAX5 |  |  | CDKN2A |  |
| PAX6 |  |  | CDV3 |  |
| PBX1 |  |  | CEBPZ |  |
| PBX3 |  |  | CELF1 |  |
| PDX1 |  |  | CELF2 |  |
| PHF8 |  |  | CELF3 |  |
| PIAS1 |  |  | CELF4 |  |
| PML |  |  | CELF5 |  |
| POLR2B |  |  | CELF6 |  |
| POLR3A |  |  | CEP170 |  |
| POLR3D |  |  | CEPT1 |  |
| POLR3G |  |  | CFL1 |  |
| POU2F1 |  |  | CGN |  |
| POU5F1 |  |  | CHCHD7 |  |
| PPARD |  |  | CHD1 |  |
| PPARG |  |  | CHERP |  |
| PRDM1 |  |  | CHORDC1 |  |
| PRKDC |  |  | CHTF8 |  |
| RAG1 |  |  | CHTOP |  |
| RARA |  |  | CIRBP |  |
| RARG |  |  | CLASP1 |  |
| RB1 |  |  | CLASP2 |  |
| RBBP5 |  |  | CLASRP |  |
| RBL2 |  |  | CLK1 |  |
| RBP2 |  |  | CLK2 |  |
| RBPJ |  |  | CLK3 |  |
| RCOR1 |  |  | CLK4 |  |
| RELA |  |  | CLP1 |  |
| RFX2 |  |  | CLTC |  |
| RFX5 |  |  | CMSS1 |  |
| RING1 |  |  | CMTR1 |  |
| RNF2 |  |  | CMTR2 |  |
| RUNX1 |  |  | CNBP |  |
| RUNX1T1 |  |  | CNN2 |  |
| RXRA |  |  | CNOT1 |  |
| RXRG |  |  | CNOT10 |  |
| RYBP |  |  | CNOT11 |  |
| SALL4 |  |  | CNOT2 |  |
| SAP30 |  |  | CNOT3 |  |
| SCML2 |  |  | CNOT4 |  |
| SETDB1 |  |  | CNOT6 |  |
| SF1 |  |  | CNOT6L |  |
| SFMBT1 |  |  | CNOT7 |  |
| SFPQ |  |  | CNOT8 |  |
| SIN3A |  |  | CNP |  |
| SIRT6 |  |  | CNTLN |  |
| SIX5 |  |  | COL5A2 |  |
| SMAD1 |  |  | COL7A1 |  |
| SMAD2 |  |  | COX4I1 |  |
| SMAD3 |  |  | COX5B |  |
| SMAD4 |  |  | CPD |  |
| SMARCA4 |  |  | CPEB1 |  |
| SMARCB1 |  |  | CPEB2 |  |
| SMARCC1 |  |  | CPEB3 |  |
| SMARCC2 |  |  | CPEB4 |  |
| SMC1A |  |  | CPS1 |  |
| SMC3 |  |  | CPSF1 |  |
| SNAI2 |  |  | CPSF2 |  |
| SNAPC2 |  |  | CPSF3 |  |
| SNAPC4 |  |  | CPSF3L |  |
| SOX17 |  |  | CPSF4 |  |
| SOX2 |  |  | CPSF4L |  |
| SOX9 |  |  | CPSF6 |  |
| SP2 |  |  | CPSF7 |  |
| SPDEF |  |  | CR925765.1 |  |
| SPIB |  |  | CRNKL1 |  |
| SRC |  |  | CRYZ |  |
| SREBF1 |  |  | CSDC2 |  |
| SREBF2 |  |  | CSDE1 |  |
| SRF |  |  | CSTF1 |  |
| SSRP1 |  |  | CSTF2 |  |
| STAT1 |  |  | CSTF2T |  |
| STAT2 |  |  | CSTF3 |  |
| STAT3 |  |  | CTAG2 |  |
| STAT4 |  |  | CTAGE5 |  |
| STAT5A |  |  | CTIF |  |
| STAT5B |  |  | CTU1 |  |
| STAT6 |  |  | CTU2 |  |
| SUMO1 |  |  | CWC15 |  |
| SUMO2 |  |  | CWC22 |  |
| SUPT5H |  |  | CWC25 |  |
| TAF1 |  |  | CWC27 |  |
| TAL1 |  |  | CWF19L1 |  |
| TAT |  |  | CWF19L2 |  |
| TBL1XR1 |  |  | CXorf23 |  |
| TBP |  |  | CYCS |  |
| TCF12 |  |  | DALRD3 |  |
| TCF21 |  |  | DAP3 |  |
| TCF7 |  |  | DARS |  |
| TCF7L1 |  |  | DARS2 |  |
| TCF7L2 |  |  | DAZ1 |  |
| TEAD1 |  |  | DAZ2 |  |
| TEAD4 |  |  | DAZ3 |  |
| TERF1 |  |  | DAZ4 |  |
| TERF2 |  |  | DAZAP1 |  |
| TET2 |  |  | DAZL |  |
| TFAP2A |  |  | DBR1 |  |
| TFAP2C |  |  | DCAF13 |  |
| THAP11 |  |  | DCP1A |  |
| TP53 |  |  | DCP1B |  |
| TP63 |  |  | DCP2 |  |
| TP73 |  |  | DCPS |  |
| TRIM28 |  |  | DDX1 |  |
| TTF2 |  |  | DDX10 |  |
| UBTF |  |  | DDX17 |  |
| USF1 |  |  | DDX18 |  |
| USF2 |  |  | DDX19A |  |
| VDR |  |  | DDX19B |  |
| VEZF1 |  |  | DDX20 |  |
| WDR5 |  |  | DDX21 |  |
| WHSC1 |  |  | DDX23 |  |
| WWTR1 |  |  | DDX24 |  |
| XBP1 |  |  | DDX25 |  |
| XRN2 |  |  | DDX26B |  |
| YAP1 |  |  | DDX27 |  |
| YY1 |  |  | DDX28 |  |
| ZBTB17 |  |  | DDX31 |  |
| ZBTB33 |  |  | DDX39A |  |
|  |  |  | DDX39B |  |
|  |  |  | DDX3X |  |
|  |  |  | DDX3Y |  |
|  |  |  | DDX4 |  |
|  |  |  | DDX41 |  |
|  |  |  | DDX42 |  |
|  |  |  | DDX43 |  |
|  |  |  | DDX46 |  |
|  |  |  | DDX47 |  |
|  |  |  | DDX49 |  |
|  |  |  | DDX5 |  |
|  |  |  | DDX50 |  |
|  |  |  | DDX51 |  |
|  |  |  | DDX52 |  |
|  |  |  | DDX53 |  |
|  |  |  | DDX54 |  |
|  |  |  | DDX55 |  |
|  |  |  | DDX56 |  |
|  |  |  | DDX58 |  |
|  |  |  | DDX59 |  |
|  |  |  | DDX6 |  |
|  |  |  | DDX60 |  |
|  |  |  | DDX60L |  |
|  |  |  | DECR1 |  |
|  |  |  | DEK |  |
|  |  |  | DENR |  |
|  |  |  | DGCR14 |  |
|  |  |  | DGCR8 |  |
|  |  |  | DHCR24 |  |
|  |  |  | DHX15 |  |
|  |  |  | DHX16 |  |
|  |  |  | DHX29 |  |
|  |  |  | DHX30 |  |
|  |  |  | DHX32 |  |
|  |  |  | DHX33 |  |
|  |  |  | DHX34 |  |
|  |  |  | DHX35 |  |
|  |  |  | DHX36 |  |
|  |  |  | DHX37 |  |
|  |  |  | DHX38 |  |
|  |  |  | DHX40 |  |
|  |  |  | DHX57 |  |
|  |  |  | DHX58 |  |
|  |  |  | DHX8 |  |
|  |  |  | DHX9 |  |
|  |  |  | DICER1 |  |
|  |  |  | DIDO1 |  |
|  |  |  | DIMT1 |  |
|  |  |  | DIP2B |  |
|  |  |  | DIS3 |  |
|  |  |  | DIS3L |  |
|  |  |  | DIS3L2 |  |
|  |  |  | DKC1 |  |
|  |  |  | DLGAP1 |  |
|  |  |  | DMXL1 |  |
|  |  |  | DNAAF2 |  |
|  |  |  | DNAH6 |  |
|  |  |  | DNAJA3 |  |
|  |  |  | DNAJC17 |  |
|  |  |  | DNAJC21 |  |
|  |  |  | DNAJC8 |  |
|  |  |  | DNAJC9 |  |
|  |  |  | DNAT1 |  |
|  |  |  | DND1 |  |
|  |  |  | DNM1L |  |
|  |  |  | DNMT1 |  |
|  |  |  | DNMT3B |  |
|  |  |  | DNTTIP2 |  |
|  |  |  | DPP10 |  |
|  |  |  | DQX1 |  |
|  |  |  | DRG1 |  |
|  |  |  | DRG2 |  |
|  |  |  | DROSHA |  |
|  |  |  | DSCAM |  |
|  |  |  | DSG1 |  |
|  |  |  | DST |  |
|  |  |  | DTD1 |  |
|  |  |  | DUS1L |  |
|  |  |  | DUS2 |  |
|  |  |  | DUS2L |  |
|  |  |  | DUS3L |  |
|  |  |  | DUS4L |  |
|  |  |  | DUSP1 |  |
|  |  |  | DUSP11 |  |
|  |  |  | DUSP23 |  |
|  |  |  | DXO |  |
|  |  |  | DYNC1H1 |  |
|  |  |  | DYNC1I2 |  |
|  |  |  | DYNLL1 |  |
|  |  |  | DZIP1 |  |
|  |  |  | DZIP1L |  |
|  |  |  | DZIP3 |  |
|  |  |  | EARS2 |  |
|  |  |  | EBNA1BP2 |  |
|  |  |  | ECH1 |  |
|  |  |  | ECT2L |  |
|  |  |  | EDC3 |  |
|  |  |  | EDC4 |  |
|  |  |  | EED |  |
|  |  |  | EEF1A1 |  |
|  |  |  | EEF1A2 |  |
|  |  |  | EEF1B2 |  |
|  |  |  | EEF1D |  |
|  |  |  | EEF1E1 |  |
|  |  |  | EEF1G |  |
|  |  |  | EEF2 |  |
|  |  |  | EEF2K |  |
|  |  |  | EEFSEC |  |
|  |  |  | EFTUD1 |  |
|  |  |  | EFTUD2 |  |
|  |  |  | EIF1 |  |
|  |  |  | EIF1AD |  |
|  |  |  | EIF1AX |  |
|  |  |  | EIF1AY |  |
|  |  |  | EIF1B |  |
|  |  |  | EIF2A |  |
|  |  |  | EIF2AK1 |  |
|  |  |  | EIF2AK2 |  |
|  |  |  | EIF2AK3 |  |
|  |  |  | EIF2AK4 |  |
|  |  |  | EIF2B1 |  |
|  |  |  | EIF2B2 |  |
|  |  |  | EIF2B3 |  |
|  |  |  | EIF2B4 |  |
|  |  |  | EIF2B5 |  |
|  |  |  | EIF2C1 |  |
|  |  |  | EIF2D |  |
|  |  |  | EIF2S1 |  |
|  |  |  | EIF2S2 |  |
|  |  |  | EIF2S3 |  |
|  |  |  | EIF2S3L |  |
|  |  |  | EIF3A |  |
|  |  |  | EIF3B |  |
|  |  |  | EIF3C |  |
|  |  |  | EIF3CL |  |
|  |  |  | EIF3D |  |
|  |  |  | EIF3E |  |
|  |  |  | EIF3G |  |
|  |  |  | EIF3H |  |
|  |  |  | EIF3I |  |
|  |  |  | EIF3J |  |
|  |  |  | EIF3K |  |
|  |  |  | EIF3L |  |
|  |  |  | EIF3M |  |
|  |  |  | EIF4A1 |  |
|  |  |  | EIF4A2 |  |
|  |  |  | EIF4A3 |  |
|  |  |  | EIF4B |  |
|  |  |  | EIF4E |  |
|  |  |  | EIF4E1B |  |
|  |  |  | EIF4E2 |  |
|  |  |  | EIF4E3 |  |
|  |  |  | EIF4ENIF1 |  |
|  |  |  | EIF4G1 |  |
|  |  |  | EIF4G2 |  |
|  |  |  | EIF4G3 |  |
|  |  |  | EIF4H |  |
|  |  |  | EIF5 |  |
|  |  |  | EIF5A |  |
|  |  |  | EIF5A2 |  |
|  |  |  | EIF5AL1 |  |
|  |  |  | EIF5B |  |
|  |  |  | EIF6 |  |
|  |  |  | ELAC1 |  |
|  |  |  | ELAC2 |  |
|  |  |  | ELAVL1 |  |
|  |  |  | ELAVL2 |  |
|  |  |  | ELAVL3 |  |
|  |  |  | ELAVL4 |  |
|  |  |  | ELOF1 |  |
|  |  |  | EMG1 |  |
|  |  |  | EML3 |  |
|  |  |  | ENDOG |  |
|  |  |  | ENDOU |  |
|  |  |  | ENDOV |  |
|  |  |  | ENO3 |  |
|  |  |  | ENOX1 |  |
|  |  |  | ENOX2 |  |
|  |  |  | EP300 |  |
|  |  |  | EPB41 |  |
|  |  |  | EPHB6 |  |
|  |  |  | EPRS |  |
|  |  |  | EPS15L1 |  |
|  |  |  | ERAL1 |  |
|  |  |  | ERCC6 |  |
|  |  |  | ERH |  |
|  |  |  | ERI1 |  |
|  |  |  | ERI2 |  |
|  |  |  | ERI3 |  |
|  |  |  | ERMP1 |  |
|  |  |  | ERN1 |  |
|  |  |  | ERN2 |  |
|  |  |  | ERO1LB |  |
|  |  |  | ERP29 |  |
|  |  |  | ESF1 |  |
|  |  |  | ESPL1 |  |
|  |  |  | ESRP1 |  |
|  |  |  | ESRP2 |  |
|  |  |  | ETF1 |  |
|  |  |  | ETFB |  |
|  |  |  | EVPLL |  |
|  |  |  | EWSR1 |  |
|  |  |  | EXO1 |  |
|  |  |  | EXOC1 |  |
|  |  |  | EXOG |  |
|  |  |  | EXOSC1 |  |
|  |  |  | EXOSC10 |  |
|  |  |  | EXOSC2 |  |
|  |  |  | EXOSC3 |  |
|  |  |  | EXOSC4 |  |
|  |  |  | EXOSC5 |  |
|  |  |  | EXOSC6 |  |
|  |  |  | EXOSC7 |  |
|  |  |  | EXOSC8 |  |
|  |  |  | EXOSC9 |  |
|  |  |  | EYS |  |
|  |  |  | EZH2 |  |
|  |  |  | FAM103A1 |  |
|  |  |  | FAM120A |  |
|  |  |  | FAM120B |  |
|  |  |  | FAM120C |  |
|  |  |  | FAM184B |  |
|  |  |  | FAM192A |  |
|  |  |  | FAM195A |  |
|  |  |  | FAM38A |  |
|  |  |  | FAM46A |  |
|  |  |  | FAM50A |  |
|  |  |  | FAM55C |  |
|  |  |  | FAM98A |  |
|  |  |  | FAM98B |  |
|  |  |  | FAM98C |  |
|  |  |  | FARP1 |  |
|  |  |  | FARS2 |  |
|  |  |  | FARSA |  |
|  |  |  | FARSB |  |
|  |  |  | FASTK |  |
|  |  |  | FASTKD1 |  |
|  |  |  | FASTKD2 |  |
|  |  |  | FASTKD3 |  |
|  |  |  | FASTKD5 |  |
|  |  |  | FAU |  |
|  |  |  | FBL |  |
|  |  |  | FBLL1 |  |
|  |  |  | FBXO17 |  |
|  |  |  | FCF1 |  |
|  |  |  | FDXACB1 |  |
|  |  |  | FGF2 |  |
|  |  |  | FIP1L1 |  |
|  |  |  | FKBP10 |  |
|  |  |  | FLNB |  |
|  |  |  | FLT1 |  |
|  |  |  | FMNL1 |  |
|  |  |  | FMR1 |  |
|  |  |  | FNBP4 |  |
|  |  |  | FRG1 |  |
|  |  |  | FRG1B |  |
|  |  |  | FTH1 |  |
|  |  |  | FTO |  |
|  |  |  | FTSJ1 |  |
|  |  |  | FTSJ2 |  |
|  |  |  | FTSJ3 |  |
|  |  |  | FUBP1 |  |
|  |  |  | FUBP3 |  |
|  |  |  | FUS |  |
|  |  |  | FXR1 |  |
|  |  |  | FXR2 |  |
|  |  |  | FYTTD1 |  |
|  |  |  | G3BP1 |  |
|  |  |  | G3BP2 |  |
|  |  |  | GADD45GIP1 |  |
|  |  |  | GAPDH |  |
|  |  |  | GAR1 |  |
|  |  |  | GARS |  |
|  |  |  | GATA4 |  |
|  |  |  | GATC |  |
|  |  |  | GCFC2 |  |
|  |  |  | GCLM |  |
|  |  |  | GDI2 |  |
|  |  |  | GEMIN2 |  |
|  |  |  | GEMIN4 |  |
|  |  |  | GEMIN5 |  |
|  |  |  | GEMIN6 |  |
|  |  |  | GEMIN7 |  |
|  |  |  | GEMIN8 |  |
|  |  |  | GFM1 |  |
|  |  |  | GFM2 |  |
|  |  |  | GIGYF2 |  |
|  |  |  | GKAP1 |  |
|  |  |  | GLE1 |  |
|  |  |  | GLG1 |  |
|  |  |  | GLTSCR2 |  |
|  |  |  | GMPPA |  |
|  |  |  | GMPR2 |  |
|  |  |  | GNL1 |  |
|  |  |  | GNL2 |  |
|  |  |  | GNL3 |  |
|  |  |  | GNL3L |  |
|  |  |  | GPATCH1 |  |
|  |  |  | GPATCH4 |  |
|  |  |  | GPATCH8 |  |
|  |  |  | GPKOW |  |
|  |  |  | GPS1 |  |
|  |  |  | GRSF1 |  |
|  |  |  | GRWD1 |  |
|  |  |  | GSDMB |  |
|  |  |  | GSDMD |  |
|  |  |  | GSPT1 |  |
|  |  |  | GSPT2 |  |
|  |  |  | GTF2F1 |  |
|  |  |  | GTF2I |  |
|  |  |  | GTF3A |  |
|  |  |  | GTPBP1 |  |
|  |  |  | GTPBP10 |  |
|  |  |  | GTPBP2 |  |
|  |  |  | GTPBP3 |  |
|  |  |  | GTPBP4 |  |
|  |  |  | GUF1 |  |
|  |  |  | H1FX |  |
|  |  |  | HABP4 |  |
|  |  |  | HADH |  |
|  |  |  | HARS |  |
|  |  |  | HARS2 |  |
|  |  |  | HBS1L |  |
|  |  |  | HDLBP |  |
|  |  |  | HEATR1 |  |
|  |  |  | HELZ |  |
|  |  |  | HELZ2 |  |
|  |  |  | HENMT1 |  |
|  |  |  | HES2 |  |
|  |  |  | HEXIM1 |  |
|  |  |  | HEXIM2 |  |
|  |  |  | HINT3 |  |
|  |  |  | HIP1 |  |
|  |  |  | HIP1R |  |
|  |  |  | HMGN1 |  |
|  |  |  | HN1L |  |
|  |  |  | HNRNPA0 |  |
|  |  |  | HNRNPA1 |  |
|  |  |  | HNRNPA1L2 |  |
|  |  |  | HNRNPA2B1 |  |
|  |  |  | HNRNPA3 |  |
|  |  |  | HNRNPAB |  |
|  |  |  | HNRNPC |  |
|  |  |  | HNRNPCL1 |  |
|  |  |  | HNRNPD |  |
|  |  |  | HNRNPDL |  |
|  |  |  | HNRNPF |  |
|  |  |  | HNRNPH1 |  |
|  |  |  | HNRNPH2 |  |
|  |  |  | HNRNPH3 |  |
|  |  |  | HNRNPK |  |
|  |  |  | HNRNPL |  |
|  |  |  | HNRNPLL |  |
|  |  |  | HNRNPM |  |
|  |  |  | HNRNPR |  |
|  |  |  | HNRNPU |  |
|  |  |  | HNRNPUL1 |  |
|  |  |  | HNRNPUL2 |  |
|  |  |  | HP1BP3 |  |
|  |  |  | HPDL |  |
|  |  |  | HPSE2 |  |
|  |  |  | HRSP12 |  |
|  |  |  | HSDL2 |  |
|  |  |  | HSPA4 |  |
|  |  |  | HSPB8 |  |
|  |  |  | HSPH1 |  |
|  |  |  | HTATSF1 |  |
|  |  |  | IARS |  |
|  |  |  | IARS2 |  |
|  |  |  | IBTK |  |
|  |  |  | ICT1 |  |
|  |  |  | IDH1 |  |
|  |  |  | IER5L |  |
|  |  |  | IFIH1 |  |
|  |  |  | IFIT1 |  |
|  |  |  | IFIT1B |  |
|  |  |  | IFIT2 |  |
|  |  |  | IFIT3 |  |
|  |  |  | IFIT5 |  |
|  |  |  | IFRD1 |  |
|  |  |  | IFT172 |  |
|  |  |  | IGF2BP1 |  |
|  |  |  | IGF2BP2 |  |
|  |  |  | IGF2BP3 |  |
|  |  |  | IGHMBP2 |  |
|  |  |  | ILF2 |  |
|  |  |  | ILF3 |  |
|  |  |  | IMP3 |  |
|  |  |  | IMP4 |  |
|  |  |  | INADL |  |
|  |  |  | INTS1 |  |
|  |  |  | INTS10 |  |
|  |  |  | INTS12 |  |
|  |  |  | INTS2 |  |
|  |  |  | INTS3 |  |
|  |  |  | INTS4 |  |
|  |  |  | INTS5 |  |
|  |  |  | INTS6 |  |
|  |  |  | INTS7 |  |
|  |  |  | INTS8 |  |
|  |  |  | INTS9 |  |
|  |  |  | INVS |  |
|  |  |  | IPO11 |  |
|  |  |  | IPO13 |  |
|  |  |  | IPO4 |  |
|  |  |  | IPO5 |  |
|  |  |  | IPO7 |  |
|  |  |  | IPO8 |  |
|  |  |  | IPO9 |  |
|  |  |  | IQGAP1 |  |
|  |  |  | IQSEC2 |  |
|  |  |  | IREB2 |  |
|  |  |  | IRF2BP2 |  |
|  |  |  | ISG20 |  |
|  |  |  | ISG20L2 |  |
|  |  |  | ISY1 |  |
|  |  |  | ITGB1 |  |
|  |  |  | JAKMIP1 |  |
|  |  |  | KARS |  |
|  |  |  | KAT8 |  |
|  |  |  | KAZN |  |
|  |  |  | KBTBD3 |  |
|  |  |  | KHDC1 |  |
|  |  |  | KHDC1L |  |
|  |  |  | KHDRBS1 |  |
|  |  |  | KHDRBS2 |  |
|  |  |  | KHDRBS3 |  |
|  |  |  | KHNYN |  |
|  |  |  | KHSRP |  |
|  |  |  | KIAA0020 |  |
|  |  |  | KIAA0101 |  |
|  |  |  | KIAA0391 |  |
|  |  |  | KIAA0430 |  |
|  |  |  | KIAA0664 |  |
|  |  |  | KIAA0907 |  |
|  |  |  | KIAA0922 |  |
|  |  |  | KIAA1429 |  |
|  |  |  | KIF11 |  |
|  |  |  | KIF18A |  |
|  |  |  | KIF1B |  |
|  |  |  | KIF2A |  |
|  |  |  | KIF2C |  |
|  |  |  | KIN |  |
|  |  |  | KLHDC4 |  |
|  |  |  | KPNA2 |  |
|  |  |  | KPNA3 |  |
|  |  |  | KPNB1 |  |
|  |  |  | KRR1 |  |
|  |  |  | KRT2 |  |
|  |  |  | KYNU |  |
|  |  |  | L1CAM |  |
|  |  |  | L1TD1 |  |
|  |  |  | LAMA2 |  |
|  |  |  | LAMB1 |  |
|  |  |  | LAMC1 |  |
|  |  |  | LARP1 |  |
|  |  |  | LARP1B |  |
|  |  |  | LARP4 |  |
|  |  |  | LARP4B |  |
|  |  |  | LARP6 |  |
|  |  |  | LARP7 |  |
|  |  |  | LARS |  |
|  |  |  | LARS2 |  |
|  |  |  | LAS1L |  |
|  |  |  | LCMT2 |  |
|  |  |  | LENG9 |  |
|  |  |  | LENG9 |  |
|  |  |  | LIMCH1 |  |
|  |  |  | LIN28A |  |
|  |  |  | LIN28B |  |
|  |  |  | LMNA |  |
|  |  |  | LONP1 |  |
|  |  |  | LRPPRC |  |
|  |  |  | LRRC40 |  |
|  |  |  | LRRC47 |  |
|  |  |  | LRRFIP1 |  |
|  |  |  | LRRFIP2 |  |
|  |  |  | LSG1 |  |
|  |  |  | LSM1 |  |
|  |  |  | LSM10 |  |
|  |  |  | LSM11 |  |
|  |  |  | LSM12 |  |
|  |  |  | LSM14A |  |
|  |  |  | LSM14B |  |
|  |  |  | LSM2 |  |
|  |  |  | LSM3 |  |
|  |  |  | LSM4 |  |
|  |  |  | LSM5 |  |
|  |  |  | LSM6 |  |
|  |  |  | LSM7 |  |
|  |  |  | LSMD1 |  |
|  |  |  | LSP1 |  |
|  |  |  | LTK |  |
|  |  |  | LUC7L |  |
|  |  |  | LUC7L2 |  |
|  |  |  | LUC7L3 |  |
|  |  |  | LUZP4 |  |
|  |  |  | MACF1 |  |
|  |  |  | MAEL |  |
|  |  |  | MAGED2 |  |
|  |  |  | MAGOH |  |
|  |  |  | MAGOHB |  |
|  |  |  | MAK16 |  |
|  |  |  | MAP1S |  |
|  |  |  | MAP7D3 |  |
|  |  |  | MAPKAPK2 |  |
|  |  |  | MAPRE1 |  |
|  |  |  | MARS |  |
|  |  |  | MARS2 |  |
|  |  |  | MAT1A |  |
|  |  |  | MATR3 |  |
|  |  |  | MAZ |  |
|  |  |  | MBNL1 |  |
|  |  |  | MBNL2 |  |
|  |  |  | MBNL3 |  |
|  |  |  | MCAM |  |
|  |  |  | MCAT |  |
|  |  |  | MCTS1 |  |
|  |  |  | MDN1 |  |
|  |  |  | MECP2 |  |
|  |  |  | MEMO1 |  |
|  |  |  | MEPCE |  |
|  |  |  | MESDC2 |  |
|  |  |  | METAP1 |  |
|  |  |  | METTL1 |  |
|  |  |  | METTL10 |  |
|  |  |  | METTL14 |  |
|  |  |  | METTL2A |  |
|  |  |  | METTL2B |  |
|  |  |  | METTL3 |  |
|  |  |  | METTL5 |  |
|  |  |  | MEX3A |  |
|  |  |  | MEX3B |  |
|  |  |  | MEX3C |  |
|  |  |  | MEX3D |  |
|  |  |  | MGST3 |  |
|  |  |  | MIF |  |
|  |  |  | MIF4GD |  |
|  |  |  | MKRN1 |  |
|  |  |  | MKRN2 |  |
|  |  |  | MKRN3 |  |
|  |  |  | MLEC |  |
|  |  |  | MLLT3 |  |
|  |  |  | MOCOS |  |
|  |  |  | MOGS |  |
|  |  |  | MOV10 |  |
|  |  |  | MOV10L1 |  |
|  |  |  | MPG |  |
|  |  |  | MPHOSPH10 |  |
|  |  |  | MPHOSPH6 |  |
|  |  |  | MRM1 |  |
|  |  |  | MRP63 |  |
|  |  |  | MRPL1 |  |
|  |  |  | MRPL10 |  |
|  |  |  | MRPL11 |  |
|  |  |  | MRPL12 |  |
|  |  |  | MRPL13 |  |
|  |  |  | MRPL14 |  |
|  |  |  | MRPL15 |  |
|  |  |  | MRPL16 |  |
|  |  |  | MRPL17 |  |
|  |  |  | MRPL18 |  |
|  |  |  | MRPL19 |  |
|  |  |  | MRPL2 |  |
|  |  |  | MRPL20 |  |
|  |  |  | MRPL21 |  |
|  |  |  | MRPL22 |  |
|  |  |  | MRPL23 |  |
|  |  |  | MRPL24 |  |
|  |  |  | MRPL27 |  |
|  |  |  | MRPL28 |  |
|  |  |  | MRPL3 |  |
|  |  |  | MRPL30 |  |
|  |  |  | MRPL32 |  |
|  |  |  | MRPL33 |  |
|  |  |  | MRPL34 |  |
|  |  |  | MRPL35 |  |
|  |  |  | MRPL36 |  |
|  |  |  | MRPL37 |  |
|  |  |  | MRPL38 |  |
|  |  |  | MRPL39 |  |
|  |  |  | MRPL4 |  |
|  |  |  | MRPL40 |  |
|  |  |  | MRPL41 |  |
|  |  |  | MRPL42 |  |
|  |  |  | MRPL43 |  |
|  |  |  | MRPL44 |  |
|  |  |  | MRPL45 |  |
|  |  |  | MRPL46 |  |
|  |  |  | MRPL47 |  |
|  |  |  | MRPL48 |  |
|  |  |  | MRPL49 |  |
|  |  |  | MRPL50 |  |
|  |  |  | MRPL51 |  |
|  |  |  | MRPL52 |  |
|  |  |  | MRPL53 |  |
|  |  |  | MRPL54 |  |
|  |  |  | MRPL55 |  |
|  |  |  | MRPL9 |  |
|  |  |  | MRPS10 |  |
|  |  |  | MRPS11 |  |
|  |  |  | MRPS12 |  |
|  |  |  | MRPS14 |  |
|  |  |  | MRPS15 |  |
|  |  |  | MRPS16 |  |
|  |  |  | MRPS17 |  |
|  |  |  | MRPS18A |  |
|  |  |  | MRPS18B |  |
|  |  |  | MRPS18B |  |
|  |  |  | MRPS18C |  |
|  |  |  | MRPS2 |  |
|  |  |  | MRPS21 |  |
|  |  |  | MRPS22 |  |
|  |  |  | MRPS23 |  |
|  |  |  | MRPS24 |  |
|  |  |  | MRPS25 |  |
|  |  |  | MRPS26 |  |
|  |  |  | MRPS27 |  |
|  |  |  | MRPS28 |  |
|  |  |  | MRPS30 |  |
|  |  |  | MRPS31 |  |
|  |  |  | MRPS33 |  |
|  |  |  | MRPS34 |  |
|  |  |  | MRPS35 |  |
|  |  |  | MRPS36 |  |
|  |  |  | MRPS5 |  |
|  |  |  | MRPS6 |  |
|  |  |  | MRPS7 |  |
|  |  |  | MRPS9 |  |
|  |  |  | MRRF |  |
|  |  |  | MRTO4 |  |
|  |  |  | MSI1 |  |
|  |  |  | MSI2 |  |
|  |  |  | MSL3 |  |
|  |  |  | MSLNL |  |
|  |  |  | MT-ATP6 |  |
|  |  |  | MT-CO2 |  |
|  |  |  | MTERF |  |
|  |  |  | MTERFD1 |  |
|  |  |  | MTERFD2 |  |
|  |  |  | MTFMT |  |
|  |  |  | MTG1 |  |
|  |  |  | MTHFSD |  |
|  |  |  | MTIF2 |  |
|  |  |  | MTIF3 |  |
|  |  |  | MTO1 |  |
|  |  |  | MTPAP |  |
|  |  |  | MTRF1 |  |
|  |  |  | MTRF1L |  |
|  |  |  | MVK |  |
|  |  |  | MVP |  |
|  |  |  | MXI1 |  |
|  |  |  | MYEF2 |  |
|  |  |  | MYH10 |  |
|  |  |  | MYH11 |  |
|  |  |  | MYO9A |  |
|  |  |  | MYST4 |  |
|  |  |  | N4BP1 |  |
|  |  |  | N6AMT1 |  |
|  |  |  | NA |  |
|  |  |  | NA |  |
|  |  |  | NAA16 |  |
|  |  |  | NAA38 |  |
|  |  |  | NAF1 |  |
|  |  |  | NANOS1 |  |
|  |  |  | NANOS2 |  |
|  |  |  | NANOS3 |  |
|  |  |  | NAP1L1 |  |
|  |  |  | NARS |  |
|  |  |  | NARS2 |  |
|  |  |  | NAT10 |  |
|  |  |  | NCBP1 |  |
|  |  |  | NCBP2 |  |
|  |  |  | NCBP2L |  |
|  |  |  | NCL |  |
|  |  |  | NCOA5 |  |
|  |  |  | NCOR2 |  |
|  |  |  | NDRG1 |  |
|  |  |  | NDRG2 |  |
|  |  |  | NEFH |  |
|  |  |  | NELFE |  |
|  |  |  | NFKB2 |  |
|  |  |  | NFX1 |  |
|  |  |  | NFXL1 |  |
|  |  |  | NGDN |  |
|  |  |  | NHP2 |  |
|  |  |  | NHP2L1 |  |
|  |  |  | NID1 |  |
|  |  |  | NIFK |  |
|  |  |  | NIP7 |  |
|  |  |  | NKRF |  |
|  |  |  | NKX6-2 |  |
|  |  |  | NLE1 |  |
|  |  |  | NLRP12 |  |
|  |  |  | NMD3 |  |
|  |  |  | NMT1 |  |
|  |  |  | NOA1 |  |
|  |  |  | NOB1 |  |
|  |  |  | NOC2L |  |
|  |  |  | NOC3L |  |
|  |  |  | NOC4L |  |
|  |  |  | NOL10 |  |
|  |  |  | NOL11 |  |
|  |  |  | NOL12 |  |
|  |  |  | NOL3 |  |
|  |  |  | NOL6 |  |
|  |  |  | NOL7 |  |
|  |  |  | NOL8 |  |
|  |  |  | NOL9 |  |
|  |  |  | NOLC1 |  |
|  |  |  | NOM1 |  |
|  |  |  | NONO |  |
|  |  |  | NOP10 |  |
|  |  |  | NOP14 |  |
|  |  |  | NOP16 |  |
|  |  |  | NOP2 |  |
|  |  |  | NOP56 |  |
|  |  |  | NOP58 |  |
|  |  |  | NOP9 |  |
|  |  |  | NOVA1 |  |
|  |  |  | NOVA2 |  |
|  |  |  | NPM1 |  |
|  |  |  | NPM2 |  |
|  |  |  | NPM3 |  |
|  |  |  | NR0B1 |  |
|  |  |  | NSA2 |  |
|  |  |  | NSRP1 |  |
|  |  |  | NSUN2 |  |
|  |  |  | NSUN3 |  |
|  |  |  | NSUN4 |  |
|  |  |  | NSUN5 |  |
|  |  |  | NSUN6 |  |
|  |  |  | NSUN7 |  |
|  |  |  | NTPCR |  |
|  |  |  | NUCB1 |  |
|  |  |  | NUDT16 |  |
|  |  |  | NUDT16L1 |  |
|  |  |  | NUDT21 |  |
|  |  |  | NUFIP1 |  |
|  |  |  | NUFIP2 |  |
|  |  |  | NUMA1 |  |
|  |  |  | NUP153 |  |
|  |  |  | NUP155 |  |
|  |  |  | NUP188 |  |
|  |  |  | NUPL2 |  |
|  |  |  | NUTF2 |  |
|  |  |  | NXF1 |  |
|  |  |  | NXF2 |  |
|  |  |  | NXF2B |  |
|  |  |  | NXF3 |  |
|  |  |  | NXF5 |  |
|  |  |  | NXT1 |  |
|  |  |  | NXT2 |  |
|  |  |  | NYNRIN |  |
|  |  |  | OAS1 |  |
|  |  |  | OAS2 |  |
|  |  |  | OAS3 |  |
|  |  |  | OASL |  |
|  |  |  | OBFC1 |  |
|  |  |  | OBSL1 |  |
|  |  |  | ODZ1 |  |
|  |  |  | OPA1 |  |
|  |  |  | OSTC |  |
|  |  |  | PA2G4 |  |
|  |  |  | PABPC1 |  |
|  |  |  | PABPC1L |  |
|  |  |  | PABPC1L2A |  |
|  |  |  | PABPC1L2B |  |
|  |  |  | PABPC3 |  |
|  |  |  | PABPC4 |  |
|  |  |  | PABPC4L |  |
|  |  |  | PABPC5 |  |
|  |  |  | PABPN1 |  |
|  |  |  | PABPN1L |  |
|  |  |  | PACSIN2 |  |
|  |  |  | PAIP1 |  |
|  |  |  | PAIP2 |  |
|  |  |  | PAIP2B |  |
|  |  |  | PAK1IP1 |  |
|  |  |  | PALLD |  |
|  |  |  | PAN2 |  |
|  |  |  | PAN3 |  |
|  |  |  | PAPD4 |  |
|  |  |  | PAPD5 |  |
|  |  |  | PAPD7 |  |
|  |  |  | PAPOLA |  |
|  |  |  | PAPOLB |  |
|  |  |  | PAPOLG |  |
|  |  |  | PARK7 |  |
|  |  |  | PARN |  |
|  |  |  | PARP1 |  |
|  |  |  | PARP12 |  |
|  |  |  | PARP4 |  |
|  |  |  | PARS2 |  |
|  |  |  | PARVA |  |
|  |  |  | PATL1 |  |
|  |  |  | PATL2 |  |
|  |  |  | PCBP1 |  |
|  |  |  | PCBP2 |  |
|  |  |  | PCBP3 |  |
|  |  |  | PCBP4 |  |
|  |  |  | PCDHAC1 |  |
|  |  |  | PCDHB4 |  |
|  |  |  | PCF11 |  |
|  |  |  | PCMT1 |  |
|  |  |  | PCNA |  |
|  |  |  | PCNT |  |
|  |  |  | PDCD11 |  |
|  |  |  | PDCD4 |  |
|  |  |  | PDCD5 |  |
|  |  |  | PDCD6 |  |
|  |  |  | PDCD6IP |  |
|  |  |  | PDCD7 |  |
|  |  |  | PDE12 |  |
|  |  |  | PDE4DIP |  |
|  |  |  | PDIA5 |  |
|  |  |  | PDIA6 |  |
|  |  |  | PEG10 |  |
|  |  |  | PELO |  |
|  |  |  | PES1 |  |
|  |  |  | PET112 |  |
|  |  |  | PFDN6 |  |
|  |  |  | PFN1 |  |
|  |  |  | PGAM1 |  |
|  |  |  | PGK1 |  |
|  |  |  | PGRMC1 |  |
|  |  |  | PHAX |  |
|  |  |  | PHB |  |
|  |  |  | PHF10 |  |
|  |  |  | PHF3 |  |
|  |  |  | PHF5A |  |
|  |  |  | PHRF1 |  |
|  |  |  | PICALM |  |
|  |  |  | PIH1D1 |  |
|  |  |  | PIH1D2 |  |
|  |  |  | PIH1D3 |  |
|  |  |  | PIN4 |  |
|  |  |  | PINX1 |  |
|  |  |  | PIP4K2C |  |
|  |  |  | PIWIL1 |  |
|  |  |  | PIWIL2 |  |
|  |  |  | PIWIL3 |  |
|  |  |  | PIWIL4 |  |
|  |  |  | PLD6 |  |
|  |  |  | PLIN3 |  |
|  |  |  | PLP2 |  |
|  |  |  | PLRG1 |  |
|  |  |  | PLS3 |  |
|  |  |  | PNKP |  |
|  |  |  | PNLDC1 |  |
|  |  |  | PNN |  |
|  |  |  | PNO1 |  |
|  |  |  | PNPT1 |  |
|  |  |  | PNRC2 |  |
|  |  |  | POLDIP3 |  |
|  |  |  | POLR1E |  |
|  |  |  | POLR2A |  |
|  |  |  | POLR2B |  |
|  |  |  | POLR2D |  |
|  |  |  | POLR2E |  |
|  |  |  | POLR2F |  |
|  |  |  | POLR2G |  |
|  |  |  | POLR2H |  |
|  |  |  | POLR2I |  |
|  |  |  | POLR2J |  |
|  |  |  | POLR2J2 |  |
|  |  |  | POLR2J3 |  |
|  |  |  | POLR2K |  |
|  |  |  | POLR2L |  |
|  |  |  | POLRMT |  |
|  |  |  | POP1 |  |
|  |  |  | POP4 |  |
|  |  |  | POP5 |  |
|  |  |  | POP7 |  |
|  |  |  | POR |  |
|  |  |  | PPA1 |  |
|  |  |  | PPAN |  |
|  |  |  | PPARGC1A |  |
|  |  |  | PPARGC1B |  |
|  |  |  | PPFIA1 |  |
|  |  |  | PPFIBP1 |  |
|  |  |  | PPIE |  |
|  |  |  | PPIH |  |
|  |  |  | PPIL3 |  |
|  |  |  | PPIL4 |  |
|  |  |  | PPM1E |  |
|  |  |  | PPME1 |  |
|  |  |  | PPP1CB |  |
|  |  |  | PPP1CC |  |
|  |  |  | PPP1R10 |  |
|  |  |  | PPP1R10 |  |
|  |  |  | PPP1R8 |  |
|  |  |  | PPRC1 |  |
|  |  |  | PPWD1 |  |
|  |  |  | PQBP1 |  |
|  |  |  | PRCC |  |
|  |  |  | PRDM2 |  |
|  |  |  | PRDX1 |  |
|  |  |  | PRDX2 |  |
|  |  |  | PRDX3 |  |
|  |  |  | PRDX6 |  |
|  |  |  | PREB |  |
|  |  |  | PRIM1 |  |
|  |  |  | PRKAR2A |  |
|  |  |  | PRKDC |  |
|  |  |  | PRKRA |  |
|  |  |  | PRMT1 |  |
|  |  |  | PRMT5 |  |
|  |  |  | PRPF18 |  |
|  |  |  | PRPF19 |  |
|  |  |  | PRPF3 |  |
|  |  |  | PRPF3 |  |
|  |  |  | PRPF31 |  |
|  |  |  | PRPF38A |  |
|  |  |  | PRPF38B |  |
|  |  |  | PRPF39 |  |
|  |  |  | PRPF4 |  |
|  |  |  | PRPF40A |  |
|  |  |  | PRPF40B |  |
|  |  |  | PRPF4B |  |
|  |  |  | PRPF6 |  |
|  |  |  | PRPF8 |  |
|  |  |  | PRR3 |  |
|  |  |  | PSIP1 |  |
|  |  |  | PSMA1 |  |
|  |  |  | PSMA6 |  |
|  |  |  | PSMD2 |  |
|  |  |  | PSMD9 |  |
|  |  |  | PSPC1 |  |
|  |  |  | PSTK |  |
|  |  |  | PTBP1 |  |
|  |  |  | PTBP2 |  |
|  |  |  | PTBP3 |  |
|  |  |  | PTCD1 |  |
|  |  |  | PTCD2 |  |
|  |  |  | PTCD3 |  |
|  |  |  | PTDSS1 |  |
|  |  |  | PTGES3 |  |
|  |  |  | PTGES3L-AARSD1 |  |
|  |  |  | PTGS1 |  |
|  |  |  | PTMS |  |
|  |  |  | PTPRF |  |
|  |  |  | PTPRM |  |
|  |  |  | PTRF |  |
|  |  |  | PTRH1 |  |
|  |  |  | PTRH2 |  |
|  |  |  | PTRHD1 |  |
|  |  |  | PUF60 |  |
|  |  |  | PUM1 |  |
|  |  |  | PUM2 |  |
|  |  |  | PURA |  |
|  |  |  | PURB |  |
|  |  |  | PURG |  |
|  |  |  | PUS1 |  |
|  |  |  | PUS10 |  |
|  |  |  | PUS3 |  |
|  |  |  | PUS7 |  |
|  |  |  | PUS7L |  |
|  |  |  | PUSL1 |  |
|  |  |  | PWP1 |  |
|  |  |  | PWP2 |  |
|  |  |  | QARS |  |
|  |  |  | QKI |  |
|  |  |  | QRSL1 |  |
|  |  |  | QSER1 |  |
|  |  |  | QTRT1 |  |
|  |  |  | QTRTD1 |  |
|  |  |  | R3HCC1 |  |
|  |  |  | R3HCC1L |  |
|  |  |  | R3HDM1 |  |
|  |  |  | R3HDM2 |  |
|  |  |  | RAB35 |  |
|  |  |  | RABGAP1 |  |
|  |  |  | RAD23B |  |
|  |  |  | RAE1 |  |
|  |  |  | RALY |  |
|  |  |  | RALYL |  |
|  |  |  | RAN |  |
|  |  |  | RANBP10 |  |
|  |  |  | RANBP17 |  |
|  |  |  | RANBP2 |  |
|  |  |  | RANBP6 |  |
|  |  |  | RAP1GAP2 |  |
|  |  |  | RAP1GDS1 |  |
|  |  |  | RAPGEF6 |  |
|  |  |  | RARS |  |
|  |  |  | RARS2 |  |
|  |  |  | RASD1 |  |
|  |  |  | RAVER1 |  |
|  |  |  | RAVER2 |  |
|  |  |  | RBBP4 |  |
|  |  |  | RBBP6 |  |
|  |  |  | RBBP7 |  |
|  |  |  | RBFOX1 |  |
|  |  |  | RBFOX2 |  |
|  |  |  | RBFOX3 |  |
|  |  |  | RBM10 |  |
|  |  |  | RBM11 |  |
|  |  |  | RBM12 |  |
|  |  |  | RBM12B |  |
|  |  |  | RBM14 |  |
|  |  |  | RBM15 |  |
|  |  |  | RBM15B |  |
|  |  |  | RBM17 |  |
|  |  |  | RBM18 |  |
|  |  |  | RBM19 |  |
|  |  |  | RBM20 |  |
|  |  |  | RBM22 |  |
|  |  |  | RBM23 |  |
|  |  |  | RBM24 |  |
|  |  |  | RBM25 |  |
|  |  |  | RBM26 |  |
|  |  |  | RBM27 |  |
|  |  |  | RBM28 |  |
|  |  |  | RBM3 |  |
|  |  |  | RBM33 |  |
|  |  |  | RBM34 |  |
|  |  |  | RBM38 |  |
|  |  |  | RBM39 |  |
|  |  |  | RBM4 |  |
|  |  |  | RBM41 |  |
|  |  |  | RBM42 |  |
|  |  |  | RBM43 |  |
|  |  |  | RBM44 |  |
|  |  |  | RBM45 |  |
|  |  |  | RBM46 |  |
|  |  |  | RBM47 |  |
|  |  |  | RBM48 |  |
|  |  |  | RBM4B |  |
|  |  |  | RBM5 |  |
|  |  |  | RBM6 |  |
|  |  |  | RBM7 |  |
|  |  |  | RBM8A |  |
|  |  |  | RBMS1 |  |
|  |  |  | RBMS2 |  |
|  |  |  | RBMS3 |  |
|  |  |  | RBMX |  |
|  |  |  | RBMX2 |  |
|  |  |  | RBMXL1 |  |
|  |  |  | RBMXL2 |  |
|  |  |  | RBMXL3 |  |
|  |  |  | RBMY1A1 |  |
|  |  |  | RBMY1B |  |
|  |  |  | RBMY1D |  |
|  |  |  | RBMY1E |  |
|  |  |  | RBMY1F |  |
|  |  |  | RBMY1J |  |
|  |  |  | RBPMS |  |
|  |  |  | RBPMS2 |  |
|  |  |  | RC3H1 |  |
|  |  |  | RC3H2 |  |
|  |  |  | RCC1 |  |
|  |  |  | RCL1 |  |
|  |  |  | RDM1 |  |
|  |  |  | RECQL |  |
|  |  |  | REPIN1 |  |
|  |  |  | REXO1 |  |
|  |  |  | REXO2 |  |
|  |  |  | REXO4 |  |
|  |  |  | RIOK1 |  |
|  |  |  | RIOK2 |  |
|  |  |  | RIOK3 |  |
|  |  |  | RNASE1 |  |
|  |  |  | RNASE10 |  |
|  |  |  | RNASE11 |  |
|  |  |  | RNASE12 |  |
|  |  |  | RNASE13 |  |
|  |  |  | RNASE2 |  |
|  |  |  | RNASE3 |  |
|  |  |  | RNASE4 |  |
|  |  |  | RNASE6 |  |
|  |  |  | RNASE7 |  |
|  |  |  | RNASE8 |  |
|  |  |  | RNASE9 |  |
|  |  |  | RNASEH1 |  |
|  |  |  | RNASEH2A |  |
|  |  |  | RNASEH2B |  |
|  |  |  | RNASEH2C |  |
|  |  |  | RNASEK |  |
|  |  |  | RNASEL |  |
|  |  |  | RNASET2 |  |
|  |  |  | RNF10 |  |
|  |  |  | RNF113A |  |
|  |  |  | RNF113B |  |
|  |  |  | RNF17 |  |
|  |  |  | RNF214 |  |
|  |  |  | RNF32 |  |
|  |  |  | RNGTT |  |
|  |  |  | RNH1 |  |
|  |  |  | RNMT |  |
|  |  |  | RNMTL1 |  |
|  |  |  | RNPC3 |  |
|  |  |  | RNPS1 |  |
|  |  |  | RP9 |  |
|  |  |  | RPA1 |  |
|  |  |  | RPF1 |  |
|  |  |  | RPF2 |  |
|  |  |  | RPGRIP1 |  |
|  |  |  | RPL10 |  |
|  |  |  | RPL10A |  |
|  |  |  | RPL10L |  |
|  |  |  | RPL11 |  |
|  |  |  | RPL12 |  |
|  |  |  | RPL13 |  |
|  |  |  | RPL13A |  |
|  |  |  | RPL14 |  |
|  |  |  | RPL15 |  |
|  |  |  | RPL17 |  |
|  |  |  | RPL18 |  |
|  |  |  | RPL18A |  |
|  |  |  | RPL19 |  |
|  |  |  | RPL21 |  |
|  |  |  | RPL22 |  |
|  |  |  | RPL22L1 |  |
|  |  |  | RPL23 |  |
|  |  |  | RPL23A |  |
|  |  |  | RPL24 |  |
|  |  |  | RPL26 |  |
|  |  |  | RPL26L1 |  |
|  |  |  | RPL27 |  |
|  |  |  | RPL27A |  |
|  |  |  | RPL28 |  |
|  |  |  | RPL29 |  |
|  |  |  | RPL3 |  |
|  |  |  | RPL30 |  |
|  |  |  | RPL31 |  |
|  |  |  | RPL32 |  |
|  |  |  | RPL34 |  |
|  |  |  | RPL35 |  |
|  |  |  | RPL35A |  |
|  |  |  | RPL36 |  |
|  |  |  | RPL36A |  |
|  |  |  | RPL36AL |  |
|  |  |  | RPL37 |  |
|  |  |  | RPL37A |  |
|  |  |  | RPL38 |  |
|  |  |  | RPL39 |  |
|  |  |  | RPL39L |  |
|  |  |  | RPL3L |  |
|  |  |  | RPL4 |  |
|  |  |  | RPL41 |  |
|  |  |  | RPL5 |  |
|  |  |  | RPL6 |  |
|  |  |  | RPL7 |  |
|  |  |  | RPL7A |  |
|  |  |  | RPL7L1 |  |
|  |  |  | RPL8 |  |
|  |  |  | RPL9 |  |
|  |  |  | RPLP0 |  |
|  |  |  | RPLP1 |  |
|  |  |  | RPLP2 |  |
|  |  |  | RPP14 |  |
|  |  |  | RPP21 |  |
|  |  |  | RPP25 |  |
|  |  |  | RPP25L |  |
|  |  |  | RPP30 |  |
|  |  |  | RPP38 |  |
|  |  |  | RPP40 |  |
|  |  |  | RPRD2 |  |
|  |  |  | RPS10 |  |
|  |  |  | RPS11 |  |
|  |  |  | RPS12 |  |
|  |  |  | RPS13 |  |
|  |  |  | RPS14 |  |
|  |  |  | RPS15 |  |
|  |  |  | RPS15A |  |
|  |  |  | RPS16 |  |
|  |  |  | RPS17 |  |
|  |  |  | RPS17L |  |
|  |  |  | RPS18 |  |
|  |  |  | RPS19 |  |
|  |  |  | RPS19BP1 |  |
|  |  |  | RPS2 |  |
|  |  |  | RPS20 |  |
|  |  |  | RPS21 |  |
|  |  |  | RPS23 |  |
|  |  |  | RPS24 |  |
|  |  |  | RPS25 |  |
|  |  |  | RPS26 |  |
|  |  |  | RPS27 |  |
|  |  |  | RPS27A |  |
|  |  |  | RPS27L |  |
|  |  |  | RPS28 |  |
|  |  |  | RPS29 |  |
|  |  |  | RPS3 |  |
|  |  |  | RPS3A |  |
|  |  |  | RPS4X |  |
|  |  |  | RPS4Y1 |  |
|  |  |  | RPS4Y2 |  |
|  |  |  | RPS5 |  |
|  |  |  | RPS6 |  |
|  |  |  | RPS7 |  |
|  |  |  | RPS8 |  |
|  |  |  | RPS9 |  |
|  |  |  | RPSA |  |
|  |  |  | RPUSD1 |  |
|  |  |  | RPUSD2 |  |
|  |  |  | RPUSD3 |  |
|  |  |  | RPUSD4 |  |
|  |  |  | RQCD1 |  |
|  |  |  | RRBP1 |  |
|  |  |  | RRNAD1 |  |
|  |  |  | RRP1 |  |
|  |  |  | RRP12 |  |
|  |  |  | RRP15 |  |
|  |  |  | RRP1B |  |
|  |  |  | RRP36 |  |
|  |  |  | RRP7A |  |
|  |  |  | RRP8 |  |
|  |  |  | RRP9 |  |
|  |  |  | RRS1 |  |
|  |  |  | RSL1D1 |  |
|  |  |  | RSL24D1 |  |
|  |  |  | RSRC1 |  |
|  |  |  | RSRC2 |  |
|  |  |  | RTCA |  |
|  |  |  | RTCB |  |
|  |  |  | RTF1 |  |
|  |  |  | RUVBL1 |  |
|  |  |  | RUVBL2 |  |
|  |  |  | RWDD4 |  |
|  |  |  | S100A7 |  |
|  |  |  | S100A8 |  |
|  |  |  | S100P |  |
|  |  |  | SAFB |  |
|  |  |  | SAFB2 |  |
|  |  |  | SAMD4A |  |
|  |  |  | SAMD4B |  |
|  |  |  | SAMHD1 |  |
|  |  |  | SAP18 |  |
|  |  |  | SARNP |  |
|  |  |  | SARS |  |
|  |  |  | SARS2 |  |
|  |  |  | SART1 |  |
|  |  |  | SART3 |  |
|  |  |  | SBDS |  |
|  |  |  | SCAF1 |  |
|  |  |  | SCAF11 |  |
|  |  |  | SCAF4 |  |
|  |  |  | SCAF8 |  |
|  |  |  | SCAMP3 |  |
|  |  |  | SDAD1 |  |
|  |  |  | SDHA |  |
|  |  |  | SEC61A1 |  |
|  |  |  | SECISBP2 |  |
|  |  |  | SECISBP2L |  |
|  |  |  | SEPSECS |  |
|  |  |  | SERBP1 |  |
|  |  |  | SERF2 |  |
|  |  |  | SETD1A |  |
|  |  |  | SETD1B |  |
|  |  |  | SETD2 |  |
|  |  |  | SETD7 |  |
|  |  |  | SETX |  |
|  |  |  | SF1 |  |
|  |  |  | SF3A1 |  |
|  |  |  | SF3A2 |  |
|  |  |  | SF3A3 |  |
|  |  |  | SF3B1 |  |
|  |  |  | SF3B14 |  |
|  |  |  | SF3B2 |  |
|  |  |  | SF3B3 |  |
|  |  |  | SF3B4 |  |
|  |  |  | SF3B5 |  |
|  |  |  | SFPQ |  |
|  |  |  | SFSWAP |  |
|  |  |  | SHMT2 |  |
|  |  |  | SHQ1 |  |
|  |  |  | SIDT1 |  |
|  |  |  | SIDT2 |  |
|  |  |  | SKIV2L |  |
|  |  |  | SKIV2L |  |
|  |  |  | SKIV2L2 |  |
|  |  |  | SLBP |  |
|  |  |  | SLC4A1AP |  |
|  |  |  | SLC4A2 |  |
|  |  |  | SLC9A3R1 |  |
|  |  |  | SLIRP |  |
|  |  |  | SLTM |  |
|  |  |  | SLU7 |  |
|  |  |  | SMAD1 |  |
|  |  |  | SMAD2 |  |
|  |  |  | SMAD3 |  |
|  |  |  | SMAD4 |  |
|  |  |  | SMAD5 |  |
|  |  |  | SMAD6 |  |
|  |  |  | SMAD7 |  |
|  |  |  | SMAD9 |  |
|  |  |  | SMG1 |  |
|  |  |  | SMG5 |  |
|  |  |  | SMG6 |  |
|  |  |  | SMG7 |  |
|  |  |  | SMG8 |  |
|  |  |  | SMG9 |  |
|  |  |  | SMN1 |  |
|  |  |  | SMN2 |  |
|  |  |  | SMNDC1 |  |
|  |  |  | SMOC1 |  |
|  |  |  | SND1 |  |
|  |  |  | SNIP1 |  |
|  |  |  | SNRNP200 |  |
|  |  |  | SNRNP25 |  |
|  |  |  | SNRNP27 |  |
|  |  |  | SNRNP35 |  |
|  |  |  | SNRNP40 |  |
|  |  |  | SNRNP48 |  |
|  |  |  | SNRNP70 |  |
|  |  |  | SNRPA |  |
|  |  |  | SNRPA1 |  |
|  |  |  | SNRPB |  |
|  |  |  | SNRPB2 |  |
|  |  |  | SNRPC |  |
|  |  |  | SNRPD1 |  |
|  |  |  | SNRPD2 |  |
|  |  |  | SNRPD3 |  |
|  |  |  | SNRPE |  |
|  |  |  | SNRPF |  |
|  |  |  | SNRPG |  |
|  |  |  | SNRPN |  |
|  |  |  | SNUPN |  |
|  |  |  | SNW1 |  |
|  |  |  | SON |  |
|  |  |  | SPAG9 |  |
|  |  |  | SPARCL1 |  |
|  |  |  | SPATS2 |  |
|  |  |  | SPATS2L |  |
|  |  |  | SPCS2 |  |
|  |  |  | SPEN |  |
|  |  |  | SPG20 |  |
|  |  |  | SQSTM1 |  |
|  |  |  | SRA1 |  |
|  |  |  | SRBD1 |  |
|  |  |  | SREK1 |  |
|  |  |  | SREK1IP1 |  |
|  |  |  | SRFBP1 |  |
|  |  |  | SRI |  |
|  |  |  | SRP14 |  |
|  |  |  | SRP19 |  |
|  |  |  | SRP54 |  |
|  |  |  | SRP68 |  |
|  |  |  | SRP72 |  |
|  |  |  | SRP9 |  |
|  |  |  | SRPK1 |  |
|  |  |  | SRPK2 |  |
|  |  |  | SRPR |  |
|  |  |  | SRRM1 |  |
|  |  |  | SRRM2 |  |
|  |  |  | SRRM3 |  |
|  |  |  | SRRM4 |  |
|  |  |  | SRRT |  |
|  |  |  | SRSF1 |  |
|  |  |  | SRSF10 |  |
|  |  |  | SRSF11 |  |
|  |  |  | SRSF12 |  |
|  |  |  | SRSF2 |  |
|  |  |  | SRSF3 |  |
|  |  |  | SRSF4 |  |
|  |  |  | SRSF5 |  |
|  |  |  | SRSF6 |  |
|  |  |  | SRSF7 |  |
|  |  |  | SRSF8 |  |
|  |  |  | SRSF9 |  |
|  |  |  | SSB |  |
|  |  |  | SSR1 |  |
|  |  |  | SSU72 |  |
|  |  |  | STAT3 |  |
|  |  |  | STAU1 |  |
|  |  |  | STAU2 |  |
|  |  |  | STRAP |  |
|  |  |  | STRBP |  |
|  |  |  | STT3B |  |
|  |  |  | SUB1 |  |
|  |  |  | SUGP1 |  |
|  |  |  | SUGP2 |  |
|  |  |  | SUMO2 |  |
|  |  |  | SUPT4H1 |  |
|  |  |  | SUPT5H |  |
|  |  |  | SUPT6H |  |
|  |  |  | SUPV3L1 |  |
|  |  |  | SURF2 |  |
|  |  |  | SURF6 |  |
|  |  |  | SUZ12 |  |
|  |  |  | SWT1 |  |
|  |  |  | SYCE1L |  |
|  |  |  | SYF2 |  |
|  |  |  | SYMPK |  |
|  |  |  | SYNCRIP |  |
|  |  |  | SYNE2 |  |
|  |  |  | TACO1 |  |
|  |  |  | TAF15 |  |
|  |  |  | TAF1L |  |
|  |  |  | TAF9 |  |
|  |  |  | TARBP1 |  |
|  |  |  | TARBP2 |  |
|  |  |  | TARDBP |  |
|  |  |  | TARS |  |
|  |  |  | TARS2 |  |
|  |  |  | TARSL2 |  |
|  |  |  | TAX1BP1 |  |
|  |  |  | TBL3 |  |
|  |  |  | TBRG4 |  |
|  |  |  | TCERG1 |  |
|  |  |  | TCF25 |  |
|  |  |  | TCOF1 |  |
|  |  |  | TDP2 |  |
|  |  |  | TDRD1 |  |
|  |  |  | TDRD10 |  |
|  |  |  | TDRD12 |  |
|  |  |  | TDRD15 |  |
|  |  |  | TDRD3 |  |
|  |  |  | TDRD5 |  |
|  |  |  | TDRD6 |  |
|  |  |  | TDRD7 |  |
|  |  |  | TDRD9 |  |
|  |  |  | TDRKH |  |
|  |  |  | TEFM |  |
|  |  |  | TEP1 |  |
|  |  |  | TERT |  |
|  |  |  | TEX10 |  |
|  |  |  | TEX13A |  |
|  |  |  | TFAM |  |
|  |  |  | TFB1M |  |
|  |  |  | TFB2M |  |
|  |  |  | TFCP2 |  |
|  |  |  | TFIP11 |  |
|  |  |  | TGS1 |  |
|  |  |  | THG1L |  |
|  |  |  | THOC1 |  |
|  |  |  | THOC2 |  |
|  |  |  | THOC3 |  |
|  |  |  | THOC5 |  |
|  |  |  | THOC6 |  |
|  |  |  | THOC7 |  |
|  |  |  | THRAP3 |  |
|  |  |  | THUMPD1 |  |
|  |  |  | THUMPD2 |  |
|  |  |  | THUMPD3 |  |
|  |  |  | TIA1 |  |
|  |  |  | TIAL1 |  |
|  |  |  | TIMM44 |  |
|  |  |  | TIPARP |  |
|  |  |  | TKTL1 |  |
|  |  |  | TLN1 |  |
|  |  |  | TLR3 |  |
|  |  |  | TLR7 |  |
|  |  |  | TLR8 |  |
|  |  |  | TMEM214 |  |
|  |  |  | TMPO |  |
|  |  |  | TNKS1BP1 |  |
|  |  |  | TNPO1 |  |
|  |  |  | TNPO2 |  |
|  |  |  | TNPO3 |  |
|  |  |  | TNRC6A |  |
|  |  |  | TNRC6B |  |
|  |  |  | TNRC6C |  |
|  |  |  | TOE1 |  |
|  |  |  | TOP1 |  |
|  |  |  | TOP2A |  |
|  |  |  | TOP3B |  |
|  |  |  | TPI1 |  |
|  |  |  | TPR |  |
|  |  |  | TRA2A |  |
|  |  |  | TRA2B |  |
|  |  |  | TRAF3IP2 |  |
|  |  |  | TRDMT1 |  |
|  |  |  | TRIM21 |  |
|  |  |  | TRIM25 |  |
|  |  |  | TRIM33 |  |
|  |  |  | TRIM56 |  |
|  |  |  | TRIM61 |  |
|  |  |  | TRIM71 |  |
|  |  |  | TRIP10 |  |
|  |  |  | TRIT1 |  |
|  |  |  | TRMT1 |  |
|  |  |  | TRMT10A |  |
|  |  |  | TRMT10B |  |
|  |  |  | TRMT10C |  |
|  |  |  | TRMT11 |  |
|  |  |  | TRMT112 |  |
|  |  |  | TRMT12 |  |
|  |  |  | TRMT13 |  |
|  |  |  | TRMT1L |  |
|  |  |  | TRMT2A |  |
|  |  |  | TRMT2B |  |
|  |  |  | TRMT44 |  |
|  |  |  | TRMT5 |  |
|  |  |  | TRMT6 |  |
|  |  |  | TRMT61A |  |
|  |  |  | TRMT61B |  |
|  |  |  | TRMU |  |
|  |  |  | TRNAU1AP |  |
|  |  |  | TRNT1 |  |
|  |  |  | TROVE2 |  |
|  |  |  | TRPT1 |  |
|  |  |  | TRUB1 |  |
|  |  |  | TRUB2 |  |
|  |  |  | TSEN15 |  |
|  |  |  | TSEN2 |  |
|  |  |  | TSEN34 |  |
|  |  |  | TSEN54 |  |
|  |  |  | TSFM |  |
|  |  |  | TSN |  |
|  |  |  | TSNAX |  |
|  |  |  | TSR1 |  |
|  |  |  | TSR2 |  |
|  |  |  | TSR3 |  |
|  |  |  | TST |  |
|  |  |  | TTC16 |  |
|  |  |  | TTF2 |  |
|  |  |  | TTK |  |
|  |  |  | TUBA1C |  |
|  |  |  | TUBB |  |
|  |  |  | TUBB2C |  |
|  |  |  | TUFM |  |
|  |  |  | TUT1 |  |
|  |  |  | TWISTNB |  |
|  |  |  | TXLNG |  |
|  |  |  | TXNDC5 |  |
|  |  |  | TXNL4A |  |
|  |  |  | TXNL4B |  |
|  |  |  | TYW1 |  |
|  |  |  | TYW3 |  |
|  |  |  | TYW5 |  |
|  |  |  | U2AF1 |  |
|  |  |  | U2AF1L4 |  |
|  |  |  | U2AF2 |  |
|  |  |  | U2SURP |  |
|  |  |  | UBA1 |  |
|  |  |  | UBA52 |  |
|  |  |  | UBAP2 |  |
|  |  |  | UBAP2L |  |
|  |  |  | UBE2O |  |
|  |  |  | UBE2T |  |
|  |  |  | UBQLN1 |  |
|  |  |  | UBTF |  |
|  |  |  | UHMK1 |  |
|  |  |  | UNK |  |
|  |  |  | UNKL |  |
|  |  |  | UPF1 |  |
|  |  |  | UPF2 |  |
|  |  |  | UPF3A |  |
|  |  |  | UPF3B |  |
|  |  |  | URB1 |  |
|  |  |  | URB2 |  |
|  |  |  | URM1 |  |
|  |  |  | USB1 |  |
|  |  |  | USP10 |  |
|  |  |  | USP16 |  |
|  |  |  | USP19 |  |
|  |  |  | USP39 |  |
|  |  |  | USP42 |  |
|  |  |  | UTP11L |  |
|  |  |  | UTP14A |  |
|  |  |  | UTP14C |  |
|  |  |  | UTP15 |  |
|  |  |  | UTP18 |  |
|  |  |  | UTP20 |  |
|  |  |  | UTP23 |  |
|  |  |  | UTP3 |  |
|  |  |  | UTP6 |  |
|  |  |  | VAMP4 |  |
|  |  |  | VARS |  |
|  |  |  | VARS2 |  |
|  |  |  | VARSL |  |
|  |  |  | VASH1 |  |
|  |  |  | VIM |  |
|  |  |  | VKORC1 |  |
|  |  |  | VPS13D |  |
|  |  |  | WAC |  |
|  |  |  | WARS |  |
|  |  |  | WARS2 |  |
|  |  |  | WBP11 |  |
|  |  |  | WBP4 |  |
|  |  |  | WDFY3 |  |
|  |  |  | WDR12 |  |
|  |  |  | WDR19 |  |
|  |  |  | WDR3 |  |
|  |  |  | WDR33 |  |
|  |  |  | WDR35 |  |
|  |  |  | WDR36 |  |
|  |  |  | WDR4 |  |
|  |  |  | WDR43 |  |
|  |  |  | WDR46 |  |
|  |  |  | WDR5 |  |
|  |  |  | WDR61 |  |
|  |  |  | WDR70 |  |
|  |  |  | WDR74 |  |
|  |  |  | WDR83 |  |
|  |  |  | WDTC1 |  |
|  |  |  | WIBG |  |
|  |  |  | WIPF3 |  |
|  |  |  | WRAP53 |  |
|  |  |  | XAB2 |  |
|  |  |  | XPNPEP3 |  |
|  |  |  | XPO1 |  |
|  |  |  | XPO4 |  |
|  |  |  | XPO5 |  |
|  |  |  | XPO6 |  |
|  |  |  | XPO7 |  |
|  |  |  | XPOT |  |
|  |  |  | XRCC1 |  |
|  |  |  | XRCC6 |  |
|  |  |  | XRN1 |  |
|  |  |  | XRN2 |  |
|  |  |  | YAP1 |  |
|  |  |  | YARS |  |
|  |  |  | YARS2 |  |
|  |  |  | YBX1 |  |
|  |  |  | YBX2 |  |
|  |  |  | YBX3 |  |
|  |  |  | YIPF3 |  |
|  |  |  | YRDC |  |
|  |  |  | YTHDC1 |  |
|  |  |  | YTHDC2 |  |
|  |  |  | YTHDF1 |  |
|  |  |  | YTHDF2 |  |
|  |  |  | YTHDF3 |  |
|  |  |  | ZC3H10 |  |
|  |  |  | ZC3H11A |  |
|  |  |  | ZC3H12A |  |
|  |  |  | ZC3H12B |  |
|  |  |  | ZC3H12C |  |
|  |  |  | ZC3H12D |  |
|  |  |  | ZC3H13 |  |
|  |  |  | ZC3H14 |  |
|  |  |  | ZC3H15 |  |
|  |  |  | ZC3H18 |  |
|  |  |  | ZC3H3 |  |
|  |  |  | ZC3H4 |  |
|  |  |  | ZC3H6 |  |
|  |  |  | ZC3H7A |  |
|  |  |  | ZC3H7B |  |
|  |  |  | ZC3H8 |  |
|  |  |  | ZC3HAV1 |  |
|  |  |  | ZC3HAV1L |  |
|  |  |  | ZC3HC1 |  |
|  |  |  | ZCCHC11 |  |
|  |  |  | ZCCHC13 |  |
|  |  |  | ZCCHC14 |  |
|  |  |  | ZCCHC17 |  |
|  |  |  | ZCCHC2 |  |
|  |  |  | ZCCHC24 |  |
|  |  |  | ZCCHC3 |  |
|  |  |  | ZCCHC5 |  |
|  |  |  | ZCCHC6 |  |
|  |  |  | ZCCHC7 |  |
|  |  |  | ZCCHC8 |  |
|  |  |  | ZCCHC9 |  |
|  |  |  | ZCRB1 |  |
|  |  |  | ZDHHC1 |  |
|  |  |  | ZFC3H1 |  |
|  |  |  | ZFHX2 |  |
|  |  |  | ZFP36 |  |
|  |  |  | ZFP36L1 |  |
|  |  |  | ZFP36L2 |  |
|  |  |  | ZFP91 |  |
|  |  |  | ZFPM2 |  |
|  |  |  | ZFR |  |
|  |  |  | ZFR2 |  |
|  |  |  | ZGPAT |  |
|  |  |  | ZMAT2 |  |
|  |  |  | ZMAT3 |  |
|  |  |  | ZMAT5 |  |
|  |  |  | ZNF106 |  |
|  |  |  | ZNF121 |  |
|  |  |  | ZNF142 |  |
|  |  |  | ZNF207 |  |
|  |  |  | ZNF239 |  |
|  |  |  | ZNF277 |  |
|  |  |  | ZNF326 |  |
|  |  |  | ZNF346 |  |
|  |  |  | ZNF385A |  |
|  |  |  | ZNF473 |  |
|  |  |  | ZNF498 |  |
|  |  |  | ZNF579 |  |
|  |  |  | ZNF593 |  |
|  |  |  | ZNF598 |  |
|  |  |  | ZNF622 |  |
|  |  |  | ZNF638 |  |
|  |  |  | ZNF668 |  |
|  |  |  | ZNF692 |  |
|  |  |  | ZNF706 |  |
|  |  |  | ZNF768 |  |
|  |  |  | ZNF831 |  |
|  |  |  | ZNFX1 |  |
|  |  |  | ZNHIT6 |  |
|  |  |  | ZRANB2 |  |
|  |  |  | ZRSR1 |  |
|  |  |  | ZRSR2 |  |
