# Supplementary material for: Construction and Validation of a Potent Epigenetic Modification-Related Prognostic Signature for Osteosarcoma Patients
Source: J Oncol. 2021 Nov 22;2021:2719172. doi: 10.1155/2021/2719172 (PMC8629625; doi:10.1155/2021/2719172)
Supplement: Supplementary Materials — Supplementary File Table S1. Epigenetic modification-related genes based on previous literature and databases. Supplementary File Table S2. 263 drugs approved by FDA or in clinical trials. Supplementary File Table S3. 53 candidate hub EMGs associated with OS through the univariate Cox regression analysis. [file 2719172.f1.zip › 2719172.f1/Table S2.docx]

| Table S2. 263 drugs approved by FDA or in clinical trials. |
| --- |
| Drug name |
| Curcumin |
| Chelerythrine |
| 3-Bromopyruvate (acid) |
| Cordycepin |
| Benzimate |
| Pimozide |
| Elesclomol |
| Wortmannin |
| geldanamycin analog |
| Elliptinium Acetate |
| Triciribine phosphate |
| BEN |
| Amonafide |
| Batracylin |
| Buthionine sulphoximine |
| Tanespimycin |
| 8-Chloro-adenosine |
| Hypothemycin |
| Fostamatinib |
| Pyrazoloacridine |
| Fenretinide |
| Dolastatin 10 |
| Staurosporine |
| Pyrazoloacridine |
| Lapachone |
| O-6-Benzylguanine |
| 7-Hydroxystaurosporine |
| 7-Hydroxystaurosporine |
| Perifosine |
| Alvocidib |
| Midostaurin |
| XK-469 |
| Triapine |
| kahalide f |
| okadaic acid |
| PD-98059 |
| Epothilone B |
| Aminoflavone |
| BN-2629 |
| LY-294002 |
| RH1 |
| XK-469 |
| 5-fluoro deoxy uridine 10mer |
| Seliciclib |
| Entinostat |
| Alvespimycin |
| 7-Tert-butyldimethylsilyl-10-hydroxycamptothecin |
| Karenitecin |
| PX-316 |
| AFP464 |
| Rebimastat |
| Imexon |
| E-7820 |
| LMP-400 |
| LMP776 |
| Lificguat |
| SR16157 |
| Dimethylaminoparthenolide |
| Selumetinib |
| BML-277 |
| Obatoclax |
| AT-13387 |
| Salinomycin |
| Itraconazole |
| XL-147 |
| Hydrastinine HCl |
| 1st Precursor Intermediate to TDP 665759 |
| (+)-JQ1 |
| Fenretinide |
| AP-26113 |
| By-Product of CUDC-305 |
| LOR-253 |
| Pelitrexol |
| Cobimetinib (isomer 1) |
| Bafetinib |
| Methotrexate |
| 6-Mercaptopurine |
| 6-Mercaptopurine |
| Nitrogen mustard |
| Allopurinol |
| Actinomycin D |
| Chlorambucil |
| Thiotepa |
| Melphalan |
| Triethylenemelamine |
| Dromostanolone Propionate |
| Acrichine |
| Fluorouracil |
| Nandrolone phenpropionate |
| Testolactone |
| Mithramycin |
| Pipobroman |
| Cyclophosphamide |
| Mitomycin |
| Floxuridine |
| Hydroxyurea |
| Uracil mustard |
| Dexamethasone Decadron |
| Dacarbazine |
| Dacarbazine |
| Vinblastine |
| Acetalax |
| Cytarabine |
| Vincristine |
| Megestrol acetate |
| tfdu |
| Procarbazine |
| Lomustine |
| Daunorubicin |
| Daunorubicin |
| Streptozocin |
| Calusterone |
| Estramustine |
| Vinblastine |
| Fluphenazine |
| Arsenic trioxide |
| Azacitidine |
| Cladribine |
| Mithramycin |
| Asparaginase |
| Ifosfamide |
| Acetalax |
| Fludarabine |
| Cisplatin |
| Isotretinoin |
| Teniposide |
| Doxorubicin |
| Fludarabine |
| Bleomycin |
| Paclitaxel |
| Decitabine |
| Mitomycin |
| Bendamustine |
| Etoposide |
| Homoharringtonine |
| Mithramycin |
| Tegafur |
| Parthenolide |
| Dexrazoxane |
| Tamoxifen |
| Pentostatin |
| Rapamycin |
| Carboplatin |
| Valrubicin |
| Idarubicin |
| Epirubicin |
| Oxaliplatin |
| Mitoxantrone |
| Cytarabine |
| Mitoxantrone |
| Fludarabine |
| Imiquimod |
| Carmustine |
| Mithramycin |
| Rapamycin |
| Clofarabine |
| Vinorelbine |
| Topotecan |
| Gemcitabine |
| bisacodyl, active ingredient of viraplex |
| Irinotecan |
| Docetaxel |
| Depsipeptide |
| Simvastatin |
| Raltitrexed |
| 7-Ethyl-10-hydroxycamptothecin |
| Bortezomib |
| Irofulven |
| Temsirolimus |
| Denileukin Diftitox Ontak |
| Pemetrexed |
| Vorinostat |
| Estramustine |
| Arsenic trioxide |
| Eribulin mesilate |
| Gefitinib |
| Erlotinib |
| Fulvestrant |
| Celecoxib |
| Zoledronate |
| Belinostat |
| Lapatinib |
| Irinotecan |
| Dasatinib |
| Everolimus |
| Pazopanib |
| Imatinib |
| Lapatinib |
| Nelfinavir |
| Nilotinib |
| Olaparib |
| Ixabepilone |
| Raloxifene |
| Abiraterone |
| Abiraterone |
| Sunitinib |
| Afatinib |
| Pazopanib |
| Olaparib |
| Depsipeptide |
| Pralatrexate |
| Pemetrexed |
| Vismodegib |
| Actinomycin D |
| Mitomycin |
| Lenvatinib |
| Nelarabine |
| Crizotinib |
| Daunorubicin |
| Digoxin |
| Ethinyl estradiol |
| Fluorouracil |
| Nitrogen mustard |
| Melphalan |
| 6-Mercaptopurine |
| Tyrothricin |
| Vinblastine |
| Cabozantinib |
| Axitinib |
| Etoposide |
| Azacitidine |
| Floxuridine |
| Trametinib |
| Palbociclib |
| Carfilzomib |
| Homoharringtonine |
| Ixazomib citrate |
| Teniposide |
| Ponatinib |
| Bleomycin |
| Paclitaxel |
| Rapamycin |
| Teniposide |
| Simvastatin |
| Belinostat |
| Doxorubicin |
| Vincristine |
| Pipamperone |
| Epirubicin |
| Idelalisib |
| Topotecan |
| Arsenic trioxide |
| 6-Mercaptopurine |
| Docetaxel |
| Vorinostat |
| Gefitinib |
| Clofarabine |
| Dasatinib |
| Irinotecan |
| Vinorelbine |
| Vandetanib |
| Cabozantinib |
| Panobinostat |
| Sonidegib |
| Vemurafenib |
| Ibrutinib |
| Alectinib |
| Dabrafenib |
| Bosutinib |
| ABT-199 |
| LDK-378 |
| LDK-378 |
| AZD-9291 |
