# Supplementary material for: Construction and Validation of a Potent Epigenetic Modification-Related Prognostic Signature for Osteosarcoma Patients
Source: J Oncol. 2021 Nov 22;2021:2719172. doi: 10.1155/2021/2719172 (PMC8629625; doi:10.1155/2021/2719172)
Supplement: Supplementary Materials — Supplementary File Table S1. Epigenetic modification-related genes based on previous literature and databases. Supplementary File Table S2. 263 drugs approved by FDA or in clinical trials. Supplementary File Table S3. 53 candidate hub EMGs associated with OS through the univariate Cox regression analysis. [file 2719172.f1.zip › 2719172.f1/Table S3.docx]

Table S3. 53 candidate hub EMGs associated with OS through the univariate Cox regression analysis

| id | HR | HR.95L | HR.95H | pvalue |
| --- | --- | --- | --- | --- |
| APOBEC3B | 0.472642 | 0.264139 | 0.84573 | 0.011591 |
| RNASE2 | 0.377203 | 0.163403 | 0.870741 | 0.022357 |
| TUBB | 8.30E-05 | 6.09E-07 | 0.011307 | 0.000179 |
| ACTN1 | 0.168463 | 0.038025 | 0.746346 | 0.019017 |
| MAGED2 | 0.091171 | 0.010136 | 0.820069 | 0.032601 |
| SAMHD1 | 0.176448 | 0.042733 | 0.728569 | 0.016501 |
| PRMT6 | 0.128942 | 0.016809 | 0.989133 | 0.048784 |
| KPNA2 | 0.124773 | 0.016732 | 0.930461 | 0.042329 |
| MYC | 12.08228 | 2.704113 | 53.98494 | 0.001105 |
| CDC42 | 0.057162 | 0.004054 | 0.805951 | 0.034027 |
| PCF11 | 10.6001 | 1.590534 | 70.64428 | 0.014708 |
| ARNTL | 0.175513 | 0.054546 | 0.564746 | 0.00352 |
| RPP40 | 0.243344 | 0.079383 | 0.745959 | 0.013407 |
| WDR12 | 4.156266 | 1.065432 | 16.21365 | 0.040245 |
| RPL37A | 10.10908 | 1.695238 | 60.28265 | 0.011107 |
| PPFIA1 | 10.25635 | 1.367884 | 76.90186 | 0.023529 |
| SNAI2 | 0.254286 | 0.071798 | 0.900604 | 0.033819 |
| TRIM21 | 0.217 | 0.072699 | 0.647727 | 0.006175 |
| PFN1 | 0.012186 | 0.000235 | 0.632181 | 0.028701 |
| MYH10 | 0.415523 | 0.201824 | 0.855492 | 0.017145 |
| DDX19A | 0.034821 | 0.003226 | 0.375901 | 0.005675 |
| PARVA | 0.110463 | 0.019183 | 0.636077 | 0.013644 |
| CLK1 | 5.217663 | 1.24076 | 21.94139 | 0.024175 |
| CNN2 | 0.160568 | 0.037405 | 0.689266 | 0.013871 |
| CPEB3 | 7.907664 | 2.867596 | 21.80612 | 6.46E-05 |
| PAPOLB | 1093.818 | 2.326071 | 514359.6 | 0.025823 |
| TERT | 4.58266 | 2.250012 | 9.333626 | 2.74E-05 |
| SAP30 | 4.389818 | 1.321972 | 14.57709 | 0.015701 |
| EHMT2 | 0.018484 | 0.001571 | 0.21748 | 0.001509 |
| EEF1D | 27.59281 | 2.787329 | 273.1516 | 0.004563 |
| EIF4E3 | 0.267818 | 0.086192 | 0.832175 | 0.022753 |
| STAT5B | 0.098091 | 0.024434 | 0.393785 | 0.00106 |
| ESRRA | 21.59048 | 2.673852 | 174.3361 | 0.003941 |
| ZCCHC8 | 7.418579 | 1.029182 | 53.4748 | 0.046756 |
| EYA2 | 0.471137 | 0.259994 | 0.853752 | 0.013092 |
| ZC3HAV1 | 20.1177 | 1.839985 | 219.9593 | 0.013908 |
| SND1 | 23.92473 | 1.791458 | 319.5122 | 0.016357 |
| MRPL48 | 11.10784 | 2.112488 | 58.40697 | 0.004468 |
| MRPS23 | 0.0903 | 0.016711 | 0.487954 | 0.005213 |
| RBM34 | 4.127965 | 1.750993 | 9.731674 | 0.001194 |
| SPARCL1 | 0.463906 | 0.219131 | 0.982101 | 0.044733 |
| TXNL4B | 0.128494 | 0.025628 | 0.644249 | 0.012614 |
| LUC7L2 | 23.77121 | 2.333239 | 242.1829 | 0.007465 |
| MAFF | 2.713947 | 1.254332 | 5.872053 | 0.011231 |
| MAFK | 6.429246 | 1.7868 | 23.13365 | 0.004394 |
| MITF | 0.250496 | 0.099239 | 0.632298 | 0.003386 |
| STK10 | 0.178692 | 0.041542 | 0.768631 | 0.020697 |
| NR2F1 | 0.392173 | 0.195873 | 0.7852 | 0.008226 |
| NEK6 | 0.250396 | 0.062891 | 0.996934 | 0.049493 |
| NXT2 | 0.318826 | 0.165487 | 0.614248 | 0.000634 |
| SMYD2 | 7.46695 | 1.441324 | 38.68343 | 0.016596 |
| TLR8 | 0.176165 | 0.037327 | 0.831413 | 0.028295 |
| TLR7 | 0.449001 | 0.209275 | 0.963334 | 0.039795 |
